# Trends and Projections in Hemorrhagic Stroke‐Related Mortality in the United States: A 1968–2030 CDC WONDER Analysis

**DOI:** 10.1002/brb3.71401

**Published:** 2026-04-14

**Authors:** Shaheer Bin Shafiq, Muhammad Salik Uddin, Muhammad Hasan, Muhammad Junaid Razzak, Muhammad Tahir, Muhammad Ahmed, Ammad Uddin, Eiman Zeeshan, Khubaib Tariq Mansoor, Hasibullah Aminpoor, Saad Ahmed Waqas

**Affiliations:** ^1^ Department of Medicine Dow University of Health Sciences Karachi Pakistan; ^2^ Dow Dental college Karachi Pakistan; ^3^ Faculty of Medicine Kabul University of Medical Sciences “Abu Ali Ibn Sina” Kabul Afghanistan

**Keywords:** age‐adjusted rates, CDC WONDER, forecasting, health disparities, hemorrhagic stroke, hypertension, mortality trends, racial disparities

## Abstract

**Background:**

Hemorrhagic stroke is a devastating cerebrovascular event with high fatality rates and major economic burden, with hypertension as the primary modifiable risk factor. This study analyzes long‐term US hemorrhagic stroke mortality trends from 1968 to 2023, identifies disparities, and projects rates through 2030.

**Methods:**

CDC WONDER data for individuals aged ≥25 years were used, identifying hemorrhagic stroke deaths via ICD codes. Age‐adjusted mortality rates (AAMRs) per 100,000 were calculated. Temporal trends were evaluated using Joinpoint regression, and projections generated with Autoregressive Integrated Moving Average (ARIMA) models.

**Results:**

From 1968 to 2023, 1,845,841 hemorrhagic stroke deaths occurred. Overall AAMRs declined from 59.86 in 1968 to 12.39 in 2023 (AAPC: –2.8%), largely reflecting improved hypertension control, though a plateau emerged from 2018 to 2021. Males consistently showed higher AAMRs than females, and Black individuals (average 32.73) had higher rates than White individuals (20.18), despite a narrowing gap. Adults ≥65 years bore the highest burden. Regionally, the South had the highest AAMRs (22.76) and slowest decline. Projections to 2030 indicate continued decline to 11.2, with persistent disparities: higher AAMRs for Black individuals (13.96) than White individuals (10.45), males (12.17) than females (10.31), and sustained elevated burden in the Midwest (11.64) and South (11.73).

**Conclusions:**

While hemorrhagic stroke mortality has fallen due to hypertension control, substantial disparities by sex, race, and geography persist and are projected to continue. Current interventions may be reaching diminishing returns, emphasizing the need for targeted, equity‐focused strategies addressing structural drivers of inequity and the rising impact of an aging population.

## Graphical abstract

Longitudinal analysis of U.S. hemorrhagic stroke mortality trends and future projections, stratified by demographic cohorts, geographic regions, and age‐adjusted mortality rates.

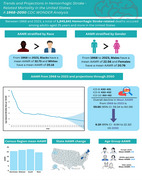

## 1 Introduction

Hemorrhagic stroke defined as intracerebral hemorrhage (ICH) or subarachnoid hemorrhage (SAH), which is a deteriorating and disabling cerebrovascular event (Rajashekar and Liang 2021), (Feigin et al. 2021). While constituting a smaller portion of all stroke cases in the United States (approximately 8% to15%), HS carries the highest case fatality rate, with ICH mortality reaching 30% to 50% within one month, resulting in severe long‐term disability for survivors (Tsao et al. 2022; Fan et al. 2024; van Asch et al. 2010).

The burden of hemorrhagic stroke is substantial and persistent. Hypertension remains the preeminent modifiable risk factor, currently affecting nearly half of all US adults (Arima et al. 2010) (U.S. Centers for Disease Control and Prevention 2025). Chronic hypertension leads to degenerative changes in the cerebral vasculature, including lipohyalinosis, fibrinoid necrosis, and microaneurysm formation, which weaken vessel walls and predispose to rupture. Elevated blood pressure also increases intracerebral shear stress and cerebral perfusion pressure, further exacerbating the risk of intracerebral hemorrhage or SAH (Medscape 2025) (Auer and Sutherland 2005).

Economically, hemorrhagic stroke places a gigantic, disproportionate economic burden. The lifetime cost of a single hemorrhagic stroke is hugely expensive, with initial estimates being over $228,000 for SAH and ICH at over $123,000 (1990 USD) (Taylor et al. 1996). This burden contributes significantly to the total US stroke cost, which exceeded $56.2 billion during 2019 to 2020, through high‐acuity medical care and substantial indirect costs from lost productivity and required long‐term care (Owolabi et al. 2022). Projections estimate that this total economic impact will escalate considerably by 2050 due to demographic shifts and the rise of key risk factors (Brown et al. 2006).

Despite hemorrhagic stroke being a major contributor to mortality, long‐term trends in hemorrhagic stroke ‐related deaths across demographic and geographic subgroups remain underexplored. Understanding these patterns is crucial for identifying disparities and guiding public health planning. The objective of the present study is to analyse hemorrhagic stroke mortality trends from 1968 to 2023 using Centers for Disease Control and Prevention's Wide‐ranging Online Data for Epidemiologic Research (CDC WONDER) data, stratified by sex, race, and geographic region, and to provide projections of hemorrhagic stroke mortality up to 2030.

## 2 Methods

### 2.1 Study Setting and Population

We used the CDC WONDER Provisional Underlying Cause of Death (UCD) database, which contains mortality data for all US residents based on death certificates. Hemorrhagic stroke deaths among individuals aged ≥25 years were identified using ICD‐8 codes 430 to 431 (1968 to 1978), ICD‐9 codes 430 to 432 (1979 to 1998), and ICD‐10 codes I60 to I62 (1999 to 2023). This study adhered to the STROBE guidelines and did not require informed consent or IRB approval, as it used publicly available, deidentified data from the CDC WONDER UCD database (Fan et al. 2024).

### 2.2 Data Abstraction

Mortality data were stratified by demographic factors, including age, sex, and race. Race was classified as White, Black, Hispanic and American Indian. Due to historical CDC WONDER reporting limitations, analysis for Hispanic and American Indian/Alaska Native populations was restricted to data from 1999 onward. Geographic variation was examined at both the regional and state levels. Regions followed U.S. Census Bureau classifications (Northeast, Midwest, South, and West), and state‐level analyses incorporated all 50 states and the District of Columbia to capture finer spatial heterogeneity.

### 2.3 Statistical Analysis

Age‐adjusted mortality rates (AAMRs) per 100,000 population were calculated using US Census population estimates and the 2000 US standard population, with 95% confidence intervals (CIs) (U.S. Census Bureau 2011) (Anderson and Rosenberg 1998). Temporal trends were analyzed using Joinpoint Regression Program version 5.4.0 (National Cancer Institute), which identified intervals of changing trends and calculated annual percentage changes (APCs) with corresponding 95% CIs (National Cancer Institute ‐ Division of Cancer Control and Population Sciences 2026). Trends were considered statistically significant at *p* < 0.05 using a two‐tailed *t*‐test. Sensitivity analyses were performed excluding pandemic years (2020–2021) to assess validity of observed trends. This study is ecological in design and does not include individual‐level data on hypertension control, obesity prevalence, anticoagulant use, or healthcare access. Therefore, temporal associations cannot establish causality.

### 2.4 Forecasting Model

To project hemorrhagic stroke mortality trends through 2030, we used an Autoregressive Integrated Moving Average (ARIMA) model. Historical mortality data from 1969 to 2015 were used for model training, and data from 2016 to 2023 were reserved for validation using time‐series cross‐validation. Model fit was assessed using the Bayesian Information Criterion (BIC) and root mean square error (RMSE), with the best‐performing specification applied to generate forecasts for 2024–2030. Projections were stratified by age and sex, and results are presented as AAMRs with 95% prediction intervals.

## 3 Results

### 3.1 Annual Trends

**FIGURE 1:**
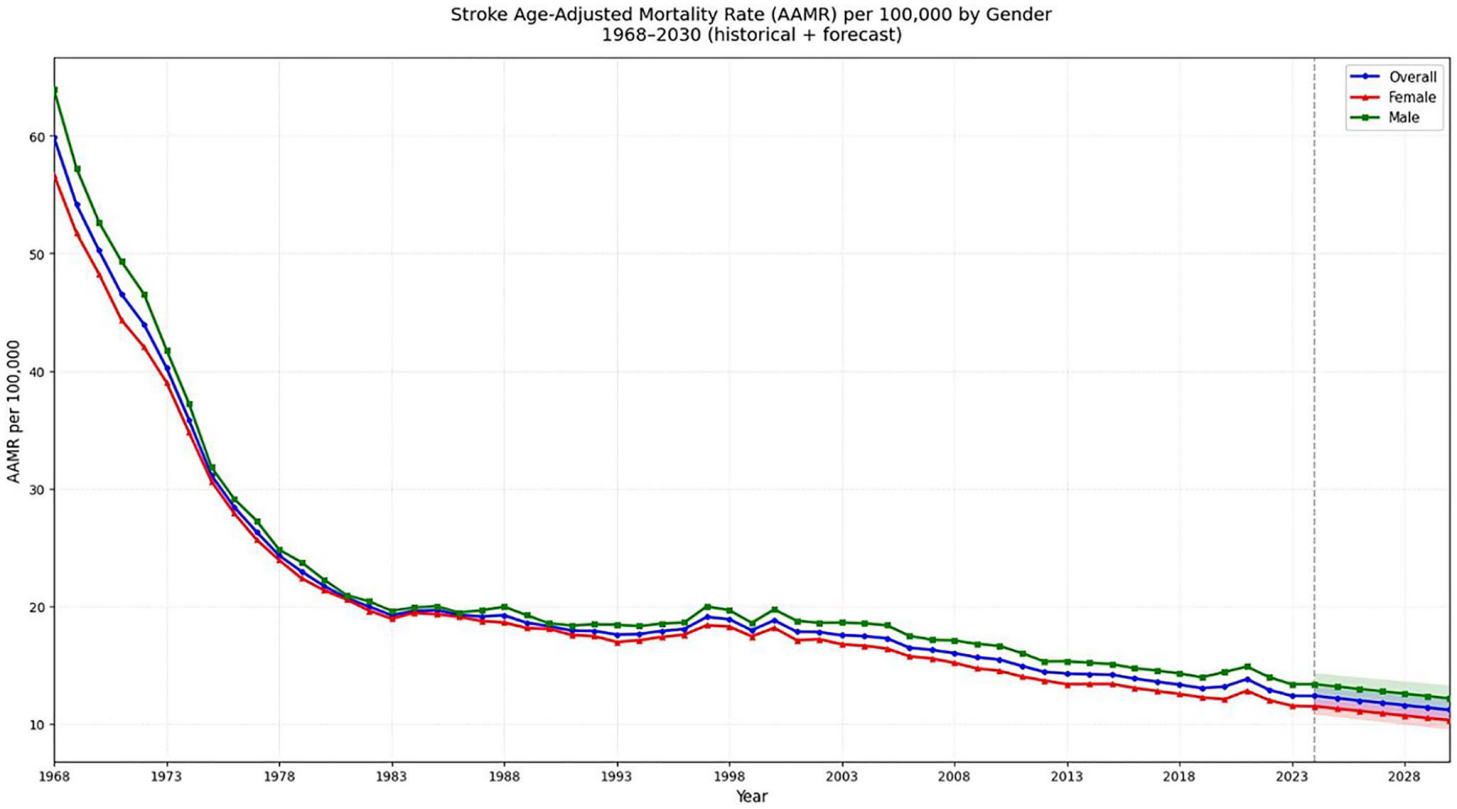
Hemorrhagic stroke AAMRs per 100,000 stratified by sex in adults in the United States, 1968–2023, and projections through 2030.

Between 1968 and 2023, a total of 1,845,841 deaths were recorded due to hemorrhagic stroke (Table S1). AAMRs declined from 59.86 (95% CI: 59.34 to 60.39) in 1968 to 12.39 (95% CI: 12.26 to 12.53) in 2023 with an AAPC of −2.8% (95% CI: −3.22 to −2.38). Mortality rates decreased from 1968 to 1973 (APC: −7.41%; 95% CI: −8.19 to −6.62), followed by a sharp drop till 1976 (APC: −11.35%; 95% CI: −15.15 to −7.37). Mortality rates experienced another decline till 1981 (APC: −6.21%; 95% CI: −7.67 to −4.74), followed by a modest decrease till 1994 (APC: −1.13%; 95% CI: −1.43 to −0.83). AAMRs experienced an inclination from 1994 to 1998 (APC: 2.18%; 95% CI: −0.16 to 4.57), and a long period of decline till 2018 (APC: −1.82%; 95% CI: −1.95 to −1.68). There was a non‐significant plateau from 2018 to 2021 (APC: 0.72%; 95% CI: −3.58 to 5.2), and a decline till 2023 (APC: −4.4%; 95% CI: −8.63 to 0.02) (Figure 1; Table 1; Tables S2, andS3).

**TABLE 1:** Annual percent change (APC) of hemorrhagic stroke related age‐adjusted mortality rates per 100,000 Adults in the United States, 1968–2023.

	Trend 1		Trend 2		Trend 3		Trend 4		Trend 5		Trend 6		Trend 7		Trend 8	
	Year	APC	Year	APC	Year	APC	Year	APC	Year	APC	Year	APC	Year	APC	Year	APC
Overall	1968–1973	−7.41(‐8.19 to ‐6.62)	1973–1976	−11.35(−15.15 to −7.37)	1976–1981	−6.21(−7.67 to −4.74)	1981–1994	−1.13(−1.43 to −0.83)	1994–1998	2.18(−0.16 to 4.57)	1998–2018	−1.82(−1.95 to −1.68)	2018–2021	0.72(−3.58 to 5.20)	2021–2023	−4.40(−8.63 to 0.02)
**Sex**																
Female	1968–1973	−7.21(−8.06 to −6.35)	1973–1977	−10.06(−12.22 to −7.92)	1977–1981	−5.46(−7.99 to −2.86)	1981–1994	−1.30(−1.61 to −0.98)	1994–1998	2.14(−0.48 to 4.83)	1998–2013	−2.04(−2.27 to −1.80)	2013–2023	−1.42(−1.81 to −1.04)	—	—
Male	1968–1980	−8.88(−9.27 to −8.49)	1980–1994	−1.06(−1.50 to −0.61)	1994–1998	2.24(−1.70 to 6.34)	1998–2023	−1.53(−1.67 to −1.38)	—	—	—	—	—	—	—	—
**Age group**																
25–44	1968–1972	−3.14(−5.12 to −1.11)	1972–1980	−6.95(−7.89 to −6.00)	1980–1997	−1.84(−2.14 to −1.55)	1997–2013	−3.01(−3.38 to −2.65)	2013–2023	0.53(−0.26 to 1.32)	—	—	—	—	—	—
45–64	1968–1972	−3.79(−5.06 to −2.50)	1972–1977	−8.03(−9.42 to −6.62)	1977–1982	−4.14(−5.83 to −2.41)	1982–1997	−1.31(−1.58 to −1.03)	1997–2017	−2.41(−2.57 to −2.24)	2017–2021	2.68(−0.35 to 5.81)	2021–2023	−4.49(−9.96 to 1.30)	—	—
65–85+	1968–1973	−8.77(−9.81 to −7.72)	1973–1976	−12.66(−17.75 to −7.24)	1976–1981	−6.83(−8.82 to −4.79)	1981–1993	−0.86(−1.32 to −0.40)	1993–2000	1.95(0.89 to 3.02)	2000–2023	−1.67(−1.80 to −1.55)	—	—	—	—
**Race**																
Black or African American	1968–1977	−9.14(−9.73 to −8.54)	1977–1981	−4.63(−8.64 to −0.45)	1981–2003	−1.27(−1.48 to −1.06)	2003–2016	−2.83(−3.30 to −2.36)	2016–2023	−0.04(−1.12 to 1.04)	—	—	—	—	—	—
White	1968–1973	−7.33(−8.22 to −6.42)	1973–1976	−11.50(−15.84 to −6.94)	1976–1981	−6.45(−8.13 to −4.74)	1981–1994	−1.27(−1.61 to −0.93)	1994–1997	3.55(−2.15 to 9.59)	1997–2023	−1.53(−1.62 to −1.43)	—	—	—	—
Hispanic	1999–2018	−2.17(−2.64 to −0.70)	2018–2021	2.74(−4.08 to 4.23)	2021–2023	−5.11(−8.57 to 0.09)	—	—	—	—	—	—	—	—	—	—
American Indian	1999–2023	−1.61(−2.10 to −1.08)	—	—	—	—	—	—	—	—	—	—	—	—	—	—
**Census region**																
Northeast	1968–1972	−6.07(−7.49 to −4.64)	1972–1977	−10.55(−12.12 to −8.95)	1977–1982	−4.68(−6.64 to −2.69)	1982–1993	−1.36(−1.88 to −0.83)	1993–2003	−0.31(−0.91 to 0.29)	2003–2023	−2.03(−2.21 to −1.84)	—	—	—	—
Midwest	1968–1973	−7.57(−8.38 to −6.76)	1973–1976	−11.13(−15.06 to −7.02)	1976–1981	−7.43(−8.97 to −5.86)	1981–1994	−1.26(−1.63 to −0.89)	1994–1997	3.53(−1.98 to 9.35)	1997–2023	−1.46(−1.56 to −1.36)	—	—	—	—
South	1968–1980	−8.98(−9.57 to −8.58)	1980–1993	−1.01(−1.51 to −0.52)	1993–2000	1.07(−0.21 to 2.36)	2000–2017	−1.90(−2.17 to −1.63)	2017–2023	−0.33(−1.49 to 0.83)	—	—	—	—	—	—
West	1968–1979	−7.17(−7.46 to −6.76)	1979–1994	−1.02(−1.33 to −0.70)	1994–1997	3.18(−2.94 to 9.68)	1997–2014	−2.32(−2.53 to −2.10)	2014–2023	−0.84(−1.36 to −0.32)	—	—	—	—	—	—


*Note*: Hispanic and American Indian populations data were restricted to data from 1999 onward.

Projections suggest AAMR could reach 11.2 (95% CI: 10.27 to 12.12) per 100,000 by 2030. Overall AAMR was predicted by weighting male and female forecasts by their projected populations for each year (Figure 1).

Our sensitivity analysis of observed and forecasted AAMRs for overall mortality (2020 to 2023) identified a pronounced spike in 2021, when observed rates increased to 13.81 (95% CI: 13.66 to 13.96), exceeding model projections and likely reflecting the effects of COVID‐19. Subsequently, the difference between observed and predicted values narrowed, with observed AAMRs decreasing to 12.39 (95% CI: 12.26 to 12.53) in 2023 and predicted rates also trending downward to 12.57 (95% CI: 11.8 to 13.35). (Figure S1).

### 3.2 Sex

Between 1968 and 2023, 827,808 males died due to hemorrhagic stroke. Males had significantly higher AAMRs than females during the study period [Males: 22.56 (95% CI: 22.19 to 22.93); Females: 20.76 (95% CI: 20.46 to 21.06)]. From 1968 to 2023, male AAMR decreased from 63.91 (95% CI: 63.06 to 64.75) to 13.37 in 2023 (95% CI: 13.15 to 13.58) with an AAPC of −2.8% (95% CI: −3.11 to −2.49). The sharpest decline in AAMR occur from 1968 to 1980 (APC: −8.88%; 95% CI: −9.27 to −8.49), and a non‐significant increase in AAMR occurred from 1994 to 1998 (APC: 2.24%; 95% CI: −1.7 to 6.34).

From 1968 and 2023, 1,018,033 deaths were recorded among females due to hemorrhagic stroke. Female AAMR experienced a decline from 56.68 (95% CI: 56.01 to 57.35) in 1968 to 11.54 (95% CI: 11.37 to 11.72) in 2023 with an AAPC of ‐2.8% (95% CI: −3.13 to −2.46). The steepest decline was observed from 1973 to 1977 (APC: −10.06%; 95% CI: −12.22 to −7.92). Mortality rates experienced an increasing trend from 1994 to 1998 (APC: 2.14%; 95% CI: −0.48 to 4.83) (Tables S2 and S3) (Figure 1).

Projections show male AAMR reaching 12.17 (95% CI: 11.04 to 13.31) and female AAMR reaching 10.31 (95% CI: 9.53 to 11.1) by 2030 (Figure 1).

### 3.3 Race

Between 1968 and 2023, 1,516,877 hemorrhagic stroke‐related deaths occurred among the White individuals and 266,262 deaths occurred among the Black population during the same period. Black individuals consistently showed higher AAMRs than whites (Blacks: 32.73 [95% CI: 31.79 to 33.67]; Whites: 20.18 [95% CI: 19.94 to 20.42]).

AAMR of White individuals decreased from 56.33 (95% CI: 55.8 to 56.87) in 1968 to 11.85 (95% CI: 11.7 to 12) in 2023 with an AAPC of −2.76% (95% CI: −3.2 to −2.33). AAMR experienced the steepest decline from 1973 to 1976 (APC: −11.5%; 95% CI: −15.84 to −6.94). An increasing trend in mortality rates was observed from 1994 to 1997 (APC: 3.55%; 95% CI: −2.15 to 9.58).

**FIGURE 2:**
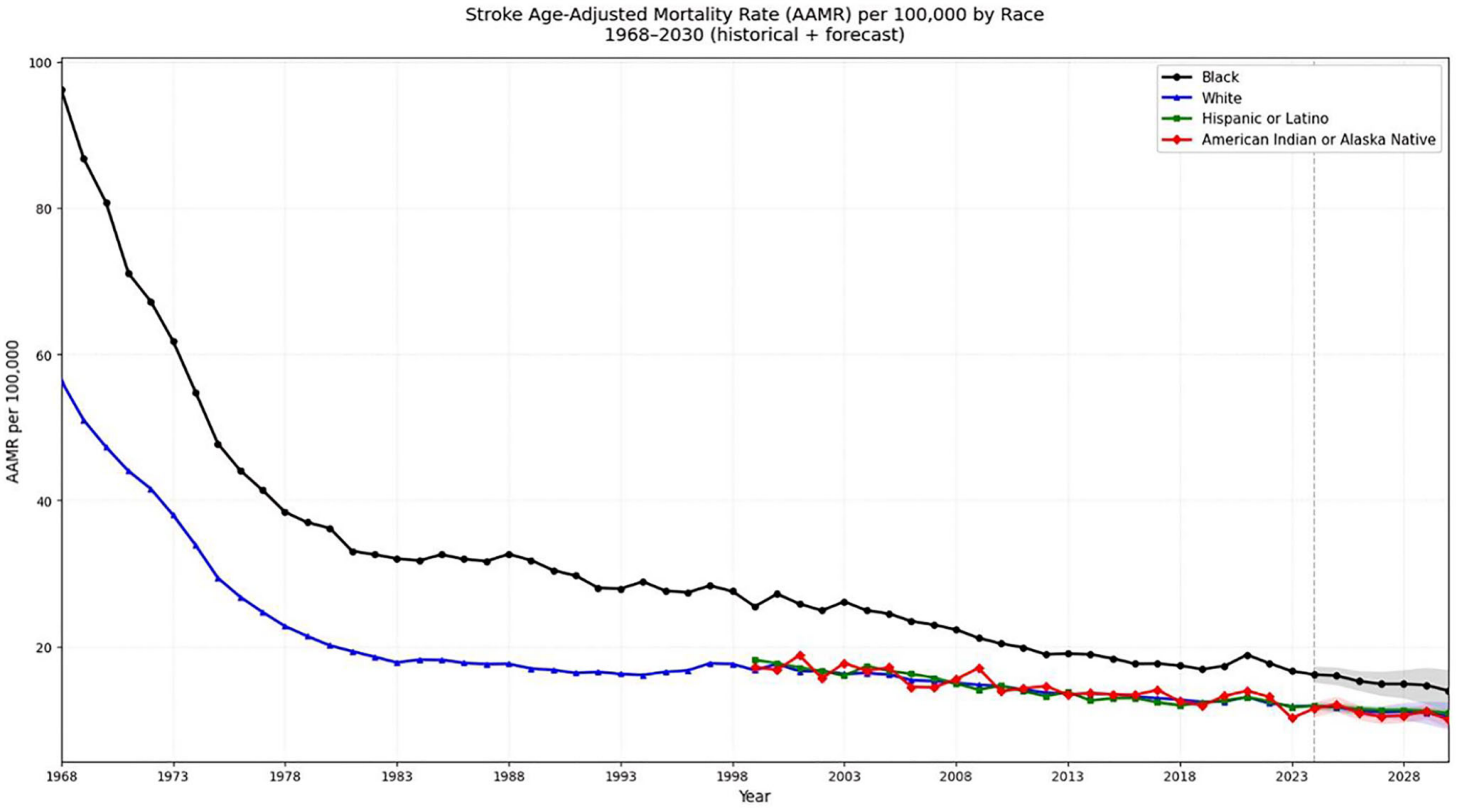
Hemorrhagic stroke AAMRs per 100,000 stratified by race in adults in the United States, 1968–2023, and projections through 2030.

AAMRs of black individuals changed from 96.14 (95% CI: 93.92 to 98.37) in 1968 to 16.63 (95% CI: 16.15 to 17.12) in 2023, corresponding to an AAPC of −3.06% (95% CI: −3.42 to −2.7). Between 1968 and 1977, AAMR experienced the steepest dip (APC: −9.14%; 95% CI: −9.73 to −8.54) (Tables S2–S5) (Figure 2).

Projections indicate that by 2030, the AAMR will decline to 10.45 (95% CI: 8.65 to 12.42) for White individuals and to 13.96 (95% CI: 11.21 to 16.83) for Black individuals (Figure 2).

AAMRs among American Indians declined from 17.15 (95% CI: 14.1 to 20.19) in 1999 to 10.22 (95% CI: 8.66 to 11.78) in 2023. Similarly, AAMRs among Hispanic individuals also decreased from 18.14 (95% CI: 17.25 to 19.03) in 1999 to 11.69 (95% CI: 11.29 to 12.09) in 2023.

Projections indicate that by 2030, the AAMR will decline to 10 (95% CI: 8.91 to 10.99) for American Indians and to 10.9 (95% CI: 10.32 to 11.52) for Hispanic individuals (Figure 2).

### 3.4 Age Group Trends

From 1968 to 2023, 129,480 deaths occurred due to hemorrhagic stroke among younger adults (25 to 44 years), 488,687 among middle‐aged adults (45 to 64 years), and 1,227,674 among older adults (≥65 years). Older adults exhibited markedly higher AAMRs than individuals in the younger age categories (Younger adults: 3.61 [95% CI: 3.47 to 3.75]; Middle‐aged adults: 15.55 [95% CI: 15.23 to 15.87]; Older adults: 74.46 [95% CI: 73.47 to 75.45]).

In younger adults, AAMR decreased from 8.53 (95% CI: 8.26 to 8.8) in 1968 to 1.89 (95% CI: 1.8 to 1.98) in 2023, corresponding to an AAPC of −2.61% (95% CI: −2.89 to −2.33). Conversely, AAMR for middle‐aged adults experienced a decline from 36.89 (95% CI: 36.3 to 37.47) in 1968 to 8.49 (95% CI: 8.29 to 8.69) in 2023 with an AAPC of −2.61% (95% CI: −2.98 to −2.24). Older adults experienced the greatest mortality burden, with AAMRs changing from 221.34 (95% CI: 218.94 to 223.73) to 44.03 (95% CI: 43.47 to 44.59) from 1968 to 2023, corresponding to an AAPC of −2.82% (95% CI: −3.23 to −2.41) (Tables S2 and S8) (Figure S2).

By 2030, projected AAMR for younger adults are 1.81 (95% CI: 1.59 to 2.02), 7.58 (95% CI: 6.57 to 8.45) for middle‐aged, and 38.35 (95% CI: 30.86 to 46.31) for older adults (Figure S2).

### 3.5 Geographic Region Trends

On average, the highest mortality rates were observed in the South (AAMR: 22.76; 95 % CI: 22.35 to 23.18), followed by the Midwest (AAMR: 22.01; 95 % CI: 21.54 to 22.47), the Northeast (AAMR: 20.79; 95 % CI: 20.3 to 21.27), and the West (AAMR: 19.44; 95 % CI: 18.91 to 19.95).

All the census regions exhibited the decreasing trend of AAMR from 1968 to 2023 with the most noticeable decline observed in Northeast (AAPC: −2.94%, 95 % CI: −3.24 to −2.64), followed by Midwest (AAPC: −2.83%, 95% CI: −3.24 to −2.43), South (AAPC: −2.75%, 95% CI: −3 to −2.49), and the least change in AAMRs was observed in the West (AAPC: −2.43%, 95% CI: −2.78 to −2.07) (Tables S2 and S7) (Figure S3).

By 2030, projected AAMR for Northeast is 9.06 (95% CI: 7.54 to 11), 11.64 (95% CI: 9.37 to 13.9) for Midwest, 11.73 (95% CI: 9.71 to 13.97) for South, and 10.25 (95% CI: 8.71 to 11.78) for West (Figure S3).

**FIGURE 3:**
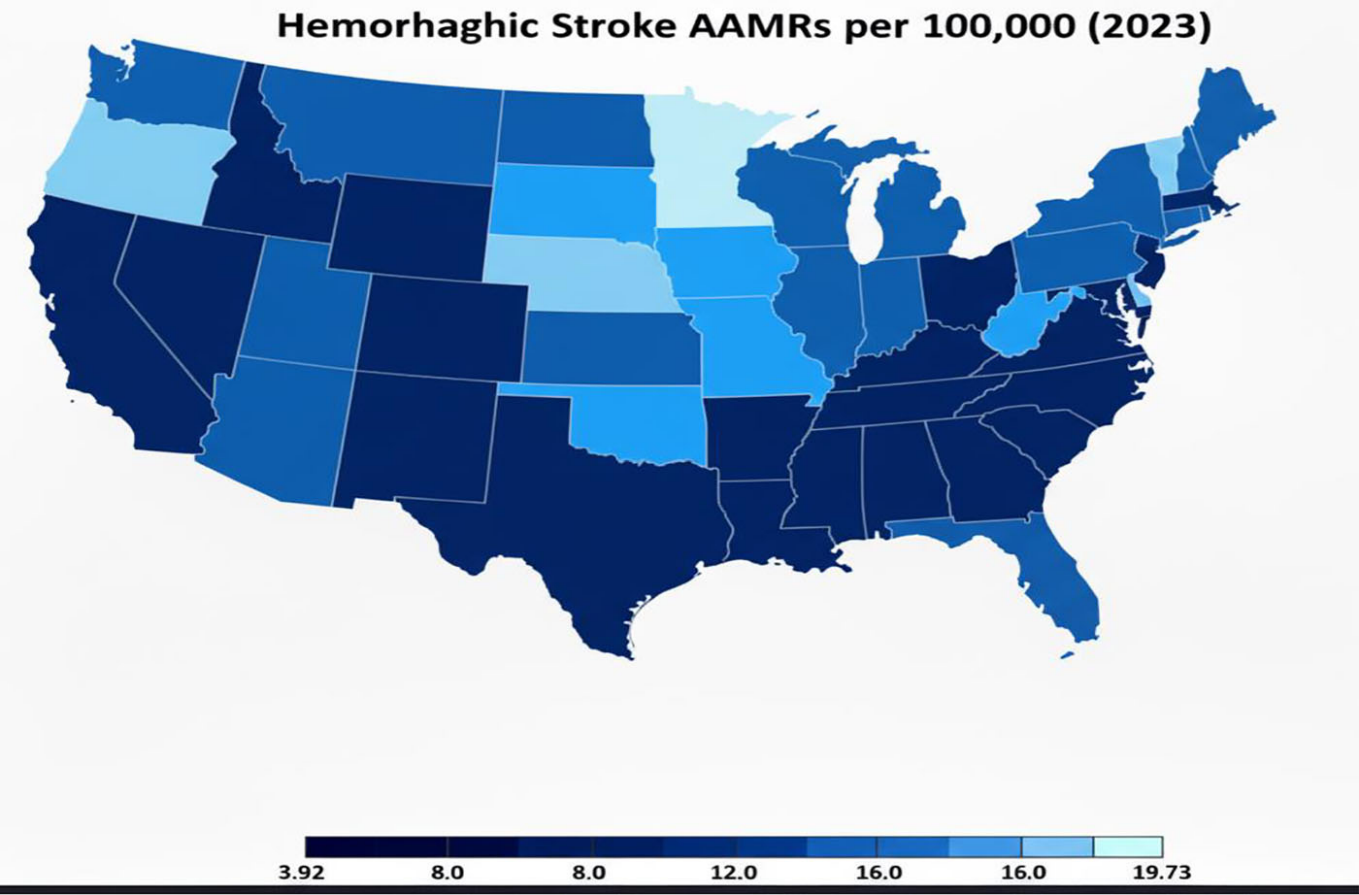
Hemorrhagic stroke AAMRs per 100,000 stratified by state in adults in the United States, 1968–2023.

States which showed the highest AAMRs in 2023 were Arkansas, Delaware and Alabama with AAMRs 19.73, 17.95, and 16.97 respectively. The states with the highest decreases in AAMRs were Maine −94.55%, Vermont −89.15% and West Virginia −88.35%. Conversely, the smallest decreases were seen in Alaska −57.19%, Delaware −59.29% and Oregon −71.45% (Figure 3; Table S6; Figure S4).

## 4 Discussion

This longitudinal analysis of hemorrhagic stroke mortality, including intracerebral (ICH) and subarachnoid (SAH) hemorrhage, reveals a profound, multi‐decade reduction in AAMRs. This progress is largely consistent with the widespread pharmacological control of hypertension, the primary modifiable driver of spontaneous ICH, though the precise contribution of individual factors cannot be determined from mortality data alone (Lackland et al. 2014) (Fewel et al. 2003). However, this success was marked by deep, persistent inequities. Our analysis confirms disproportionate mortality burdens among Black individuals and males, alongside consistently elevated rates in the South that mirror the well‐documented “Stroke Belt” (Howard and Howard 2020). Despite a recent period of instability, our model projects a continued downward trend through 2030. The following discussion will therefore seek to interpret the drivers of these trends, explore the causes of observed disparities, and contextualize our near‐term projections.

The historical trend in hemorrhagic stroke mortality reflected distinct epochs of public health and clinical shifts. The sharpest declines (1968 to 1981) aligned with the modern antihypertensive era, catalyzed by the 1972 National High Blood Pressure Education Program and effective pharmacotherapies (Jones and Hall 2002) (Kawachi and Wilson 1990). This decline moderated from 1981 to 1994, suggesting severe, untreated hypertension was largely addressed. A brief increase followed (1994 to 1998), coinciding with rising obesity and diabetes epidemics that counteracted blood pressure control gains (Mokdad et al. 2001) (Pezzini et al. 2013). A renewed decline (1998 to 2018) likely reflected clinical advancements, particularly standardized acute hemorrhage management in Primary Stroke Centers and neuro‐critical care (Lichtman et al. 2011). However, progress stalled between 2018 and 2019, prior to the COVID‐19 pandemic, suggesting that factors independent of pandemic‐related disruptions—including a potential ceiling in hypertension control, advancing cardiometabolic epidemics, and rising anticoagulation‐associated ICH in an aging population — had already begun to attenuate mortality gains (Delago et al. 2022) (Morotti and Goldstein 2020). The subsequent pandemic period (2020 to 2021) was associated with documented healthcare disruptions and worsened in‐hospital outcomes for hemorrhagic stroke patients, which may have further compounded this stagnation (Bako et al. 2024). Consequently, the subsequent downturn observed from 2021 to 2023 requires cautious interpretation and cannot be confidently attributed to a single mechanism; it may reflect post‐pandemic healthcare recovery, statistical volatility, or a genuine resumption of the prior declining trend.

Our analysis confirms a durable sex disparity, with males exhibiting consistently higher AAMRs. This aligns with literature attributing elevated male risk to a greater burden of hypertension, particularly at younger ages, alongside historically higher smoking and alcohol consumption (Connelly et al. 2022) (Gokhale et al. 2015). Conversely, the higher absolute death toll among women reflects demographics rather than age‐specific risk. Longer female life expectancy increases the population at risk in advanced age, where incidence peaks (Reeves et al. 2008). Most notably, historical trends were parallel. Both sexes experienced nearly identical declines, suggesting that public health interventions, including blood pressure control, were associated with equitable benefits across sexes. This parallelism extended to negative trends, including the mid‐1990s mortality increase associated with emerging obesity and diabetes epidemics (Mokdad et al. 2001; Pezzini et al. 2013).

Beyond sex disparities, our analysis confirms a pronounced, persistent inequity between Black and White individuals. Black individuals experienced consistently higher AAMRs, reflecting elevated population‐level risk for both major hemorrhagic stroke subtypes, ICH and SAH (Broderick et al. 1992). This burden is likely multifactorial, associated with the higher prevalence, severity, and earlier onset of hypertension, compounded by social determinants of health, including structural racism (Muntner et al. 2020) (Churchwell et al. 2020). Conversely, lower mortality among White individuals reflects systemic advantages. Historically better access to primary care and higher insurance rates facilitate earlier detection and more effective, sustained hypertension management (Aggarwal et al. 2021) (Smith 2025). Consequently, the gap in hypertension control, rather than prevalence alone, may be a key contributor to divergent mortality outcomes. Encouragingly, the absolute mortality gap narrowed substantially over time. This convergence was driven by a faster overall decline among Black individuals, particularly during the early hypertension control era, consistent with public health efforts delivering meaningful benefits to this high‐risk population (Lackland et al. 2014). However, this progress was insufficient to achieve equity. A significant mortality gap persisted at the end of the study period. This gap represents a critical, unresolved failure of public health and demonstrates that universal interventions alone are insufficient to overcome these deeply entrenched systemic disadvantages. Expanding beyond Black and White individuals, American Indian/Alaska Native and Hispanic populations also carry substantial hemorrhagic stroke mortality burdens, shaped by distinct risk profiles including lower hypertension control rates, language and cultural barriers to care, and disparities in access to acute and post‐acute stroke services (Noushad et al. 2025).

While racial disparities highlight modifiable systemic inequities, our analysis by age confirms the dominant role of a non‐modifiable biological factor. Advanced age is the most powerful determinant of hemorrhagic stroke mortality, with older adults (65+) exhibiting exponentially higher AAMRs than younger groups. This expected gradient reflects the fundamental mechanisms of vascular aging, the cumulative, lifelong burden of hypertension, and the increased prevalence of complicating factors (O'Donnell et al. 2016). A critical complicating factor in this older cohort is the rising prevalence of atrial fibrillation, which necessitates oral anticoagulation and creates a parallel iatrogenic risk of ICH (Morotti and Goldstein 2020). Despite these distinct risk profiles, the most striking finding is that all three age groups experienced a remarkably similar average annual rate of decline. This powerful cohort effect is consistent with foundational public health interventions, particularly widespread hypertension management, conferring universal benefits across all ages (Lackland et al. 2014) (Williamson et al. 2016). However, this historical success contrasts with recent literature documenting increasing ICH incidence among younger populations. This trend, associated with earlier onset obesity and diabetes, threatens to reverse 20th‐century mortality gains (Kissela et al. 2012).

Complementing the demographic disparities observed, our analysis also confirmed a durable geographic clustering of hemorrhagic stroke mortality. We found that the Southern United States had the highest average AAMRs across the entire study period, a finding for hemorrhagic stroke that mirrors the well‐documented and previously mentioned “Stroke Belt” phenomenon observed for total stroke mortality (Howard and Howard 2020). This enduring regional disparity is recognized as a complex, multifactorial problem that is deeply rooted in a higher population‐level prevalence of key hemorrhagic stroke risk factors, including hypertension, obesity, and diabetes (Howard et al. 2005). These clinical factors are often compounded by regional socioeconomic challenges, including a distinct dietary pattern high in sodium and processed foods, disparities in healthcare infrastructure, lower rates of Medicaid expansion, reduced access to certified stroke centers, and higher social vulnerability indices (Judd et al. 2013) (Mullen et al. 2014). Perhaps more concerning than the high baseline is our finding that the rate of mortality declines in the South, while substantial, was slower than in the Northeast and Midwest. This differential in progress suggests that the life‐saving benefits of national hypertension control gains were not equitably distributed, likely blunted in the South by the entrenched nature of these risks, persistent barriers to primary and acute specialty care, and structural gaps in stroke system organization (Mullen et al. 2014). Projections to 2030 reveal a notable convergence, with the South and Midwest expected to carry nearly equivalent mortality burdens, in contrast to the Northeast, which is projected to achieve the lowest regional. This convergence likely reflects the South's faster recent declines from a historically high baseline, while the Midwest's comparatively slower progress suggests emerging cardiometabolic risk trends and structural barriers that warrant targeted regional intervention.

Our ARIMA‐based forecast, extrapolating from historical temporal trends, projects a continued decline in the overall AAMR, estimated to reach 11.2 per 100,000 (95% CI: 10.27 to 12.12) by 2030. Importantly, these projections reflect mortality rates rather than stroke incidence; declining mortality may reflect reduced stroke occurrence due to prevention, improved acute survival due to advances in neurocritical care, or both—distinctions with fundamentally different policy implications that cannot be resolved from death certificate data alone. Notably, our sensitivity analysis identified a pronounced spike in observed mortality in 2021, exceeding model projections and consistent with COVID‐19‐related healthcare disruptions, after which observed and predicted rates reconverged by 2023, lending support to the near‐term projection trajectory. Perhaps more consequential than the aggregate trend is the projected persistence of historical inequities, suggesting a “status quo” trajectory for health disparities. Specifically, the central estimates indicate that established mortality gaps will endure. The AAMR for Black individuals is forecast to remain higher than for White individuals, and the male mortality rate is projected to continue to exceed the female rate. Geographically, the spatial clustering of mortality is also forecast to persist, with the Midwest and South remaining the regions with the highest mortality burden. The wide CIs observed across subgroup projections—most notably for older adults, where the 2030 estimate of 38.35 carries a CI of 30.86 to 46.31—reflect the substantial statistical uncertainty introduced by recent trend instability, and long‐term projections beyond 2030 should be interpreted with particular caution given intervals that include implausible negative values.

The primary implication of these projections is that foundational public health interventions, particularly primary hypertension control, may have reached a point of diminishing returns in reducing mortality rates. While associated with substantial aggregate mortality reductions, these strategies appear insufficient to eliminate entrenched disparities. Thus, absent novel interventions targeting systemic drivers of inequity, these mortality gaps are unlikely to resolve autonomously. Sustaining progress requires a multi‐pronged strategy. Clinicians must prioritize overcoming therapeutic inertia, particularly in high‐risk populations, and conduct vigilant risk‐benefit assessments regarding oral anticoagulation to mitigate iatrogenic ICH (Judd et al. 2013) (Frontera et al. 2016). Policymakers must renew funding for community‐based screening, especially in the “Stroke Belt,” and implement policies addressing the social determinants of health driving racial disparities (Havranek et al. 2015). Researchers are mandated to clarify specific disparity drivers, distinguish the relative contributions of stroke prevention versus improved acute survival to observed mortality declines, and identify prevention strategies beyond hypertension control. These steps are vital because the aging U.S. population will increase the absolute number of high‐risk adults. Consequently, despite declining rates, the total clinical and economic burden of hemorrhagic stroke will remain a significant public health challenge (Ovbiagele et al. 2013).

### 4.1 Limitations

It is important to acknowledge several limitations inherent in this analysis. First, our reliance on CDC WONDER UCD data are subject to potential misclassification of hemorrhagic stroke subtypes, a challenge compounded by the need to bridge mortality definitions across three different ICD revisions (ICD‐8, −9, and −10) over the 55‐year study period. More significantly, death certificate data lacks crucial clinical detail. It cannot differentiate between hemorrhagic stroke etiologies (e.g., hypertensive ICH vs. amyloid angiopathy or aneurysmal SAH), nor can it provide data on the prevalence or control of key risk factors, such as anticoagulation status or medication adherence. Our interpretations of the drivers behind the trends are therefore based on ecological associations with published literature, not on individual‐level data. Furthermore, although we expanded our analysis to include Hispanic and American Indian/Alaska Native populations from 1999 onward, data on Asian American and other racial and ethnic groups were insufficient for robust trend analysis; thus, our findings are not fully generalizable across all racial and ethnic groups in the United States. Additionally, the combination of ICH and SAH into a single hemorrhagic stroke category, necessitated by ICD coding constraints across revisions, may obscure subtype‐specific trends. In particular, SAH—which disproportionately affects younger adults and carries a distinct etiology centered on aneurysmal rupture rather than hypertension—may be partially masked by the dominant ICH signal, limiting the interpretability of age‐specific findings.

Second, our ARIMA forecast has inherent limitations. Like all time‐series models, it assumes that past trends are predictive of the future and cannot account for novel external shocks, such as the development of transformative new therapies or future health crises. This is particularly salient for our study, as the recent historical volatility, including the 2018 to 2021 plateau and the subsequent 2021 to 2023 downturn, resulted in the exceptionally wide CIs observed across subgroup projections, most notably for older adults, where CIs include implausible negative values. To address this, CIs were truncated at zero where applicable; however, future modeling efforts should consider log‐transformation prior to ARIMA fitting to ensure non‐negative projections across all subgroups. This high statistical uncertainty suggests that while the central estimates are informative for a “status quo” scenario, the near‐term AAMR trajectory through 2030 remains sensitive to these recent, unstable trends, and projections should be interpreted as a “status quo” scenario rather than a definitive forecast.

## 5 Conclusion

Long‐term mortality trends for hemorrhagic stroke reveal a dual narrative: the monumental success of 20th‐century public health in reducing death rates, juxtaposed with a persistent failure to resolve deep inequities. The historical decline, largely consistent with the benefits of hypertension control, was universal but insufficient to close the stark gaps affecting Black individuals or those in the “Stroke Belt.” Looking forward, our forecast serves as a critical warning, projecting a “status quo” future where these racial and geographic disparities are estimated to persist to 2030. This stagnation in equity, coupled with a rapidly aging population, ensures the absolute clinical and economic burden of this condition will remain a significant national challenge. Having approached the limits of foundational interventions, future progress will be defined by our ability to deploy targeted, equity‐focused strategies that address the structural and social determinants underlying these persistent disparities.

## Author Contributions

**S.B.S**: conceptualization, project administration, methodology, data curation, writing – original draft, formal analysis. **M.S.U**: writing – original draft, methodology, formal analysis. **M.H**: supervision, investigation, writing – review and editing, validation. **M.J.R**: data curation, formal analysis, software, writing – review and editing. **M.T**: validation, visualization, writing – review and editing. **M.A**: methodology, investigation, writing – review and editing, resources. **A.U**: conceptualization, supervision, writing – review and editing, formal analysis, writing – original draft. **E.Z**: data curation, visualization, investigation, writing – review and editing. **K.T.M**: data curation, visualization, writing – review and editing. **H.A**: methodology, data curation, writing – original draft, formal analysis. **S.A.W**: data curation, visualization, investigation. All authors have read and approved the final manuscript.

## Funding

The authors have nothing to report.

## Conflicts of Interest

The authors declare no conflicts of interest.

## Supporting information




**Supplementary Materials**: brb371401‐sup‐0001‐SuppMat.docx

## Data Availability

The data supporting the findings of this study were obtained from the CDC WONDER online database (Centers for Disease Control and Prevention Wide‐ranging Online Data for Epidemiologic Research). The datasets used and analyzed during the current study are publicly available and can be accessed at [CDC WONDER] (https://wonder.cdc.gov).
