# Supplementary material for: Trends and Projections in Hemorrhagic Stroke‐Related Mortality in the United States: A 1968–2030 CDC WONDER Analysis
Source: Brain Behav. 2026 Apr 14;16(4):e71401. doi: 10.1002/brb3.71401 (PMC13080105; doi:10.1002/brb3.71401)
Supplement: Supplementary file 1 — Supplementary Materials: brb371401‐sup‐0001‐SuppMat.docx [file BRB3-16-e71401-s001.docx]

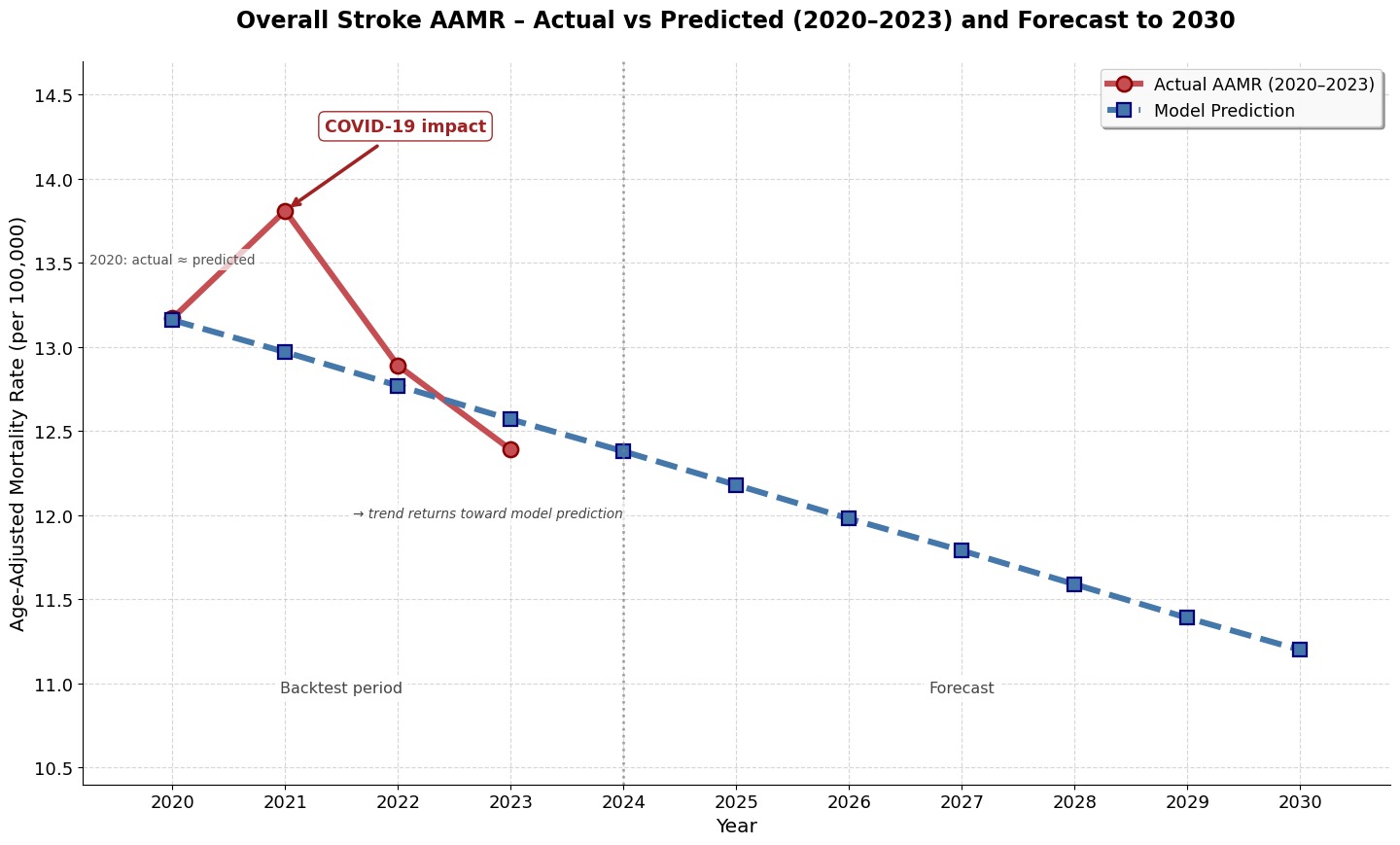


**Supplemental Figure 1.** Sensitivity Analysis and Model Validation Backtesting of stroke AAMR projections against observed 2020–2023 mortality data, accounting for COVID-19-related deviations.


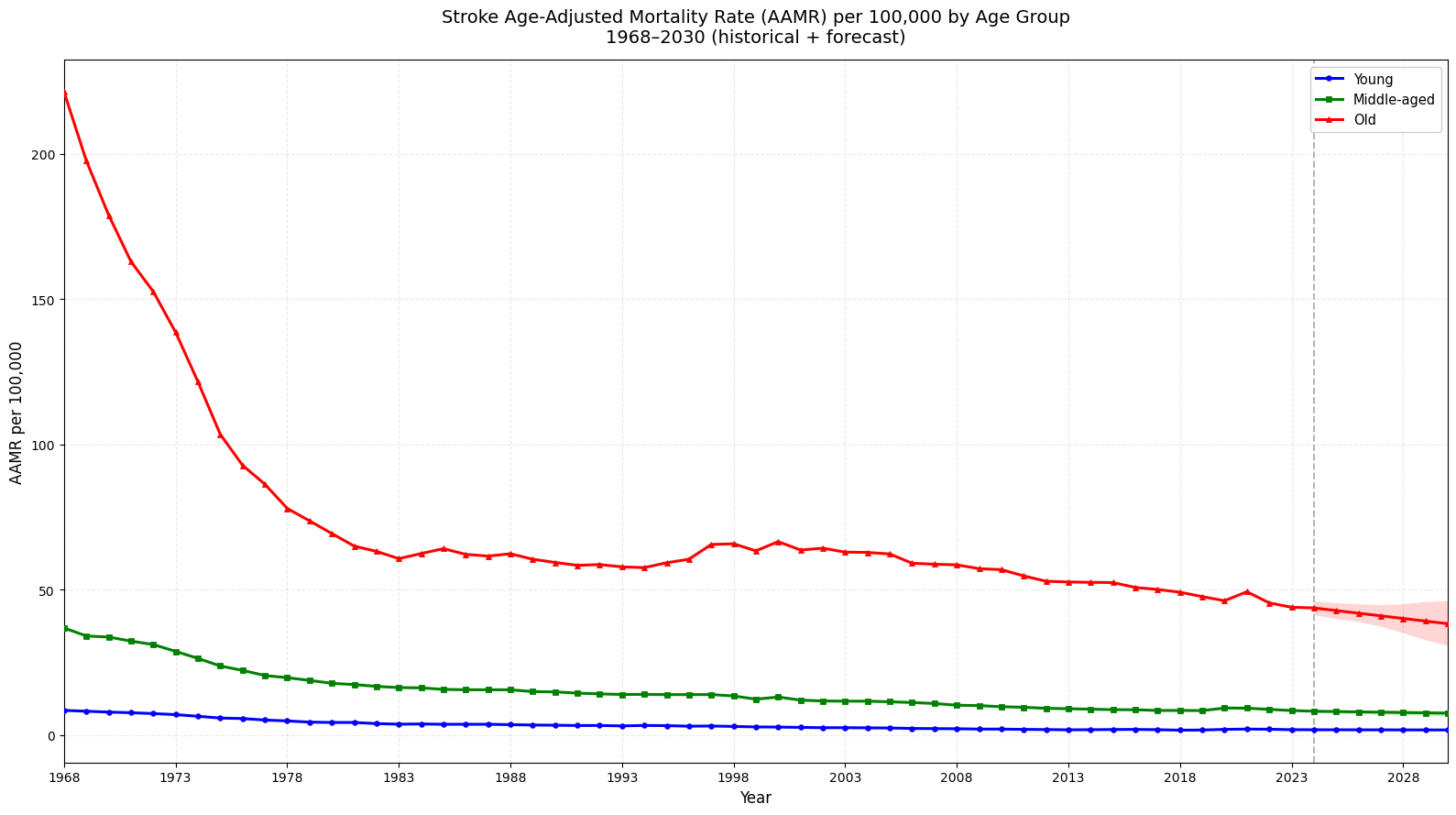


**Supplemental Figure 2.** Hemorrhagic Stroke AAMRs per 100,000 stratified by age group in adults in the United States, 1968­­–2023 and projections through 2030.


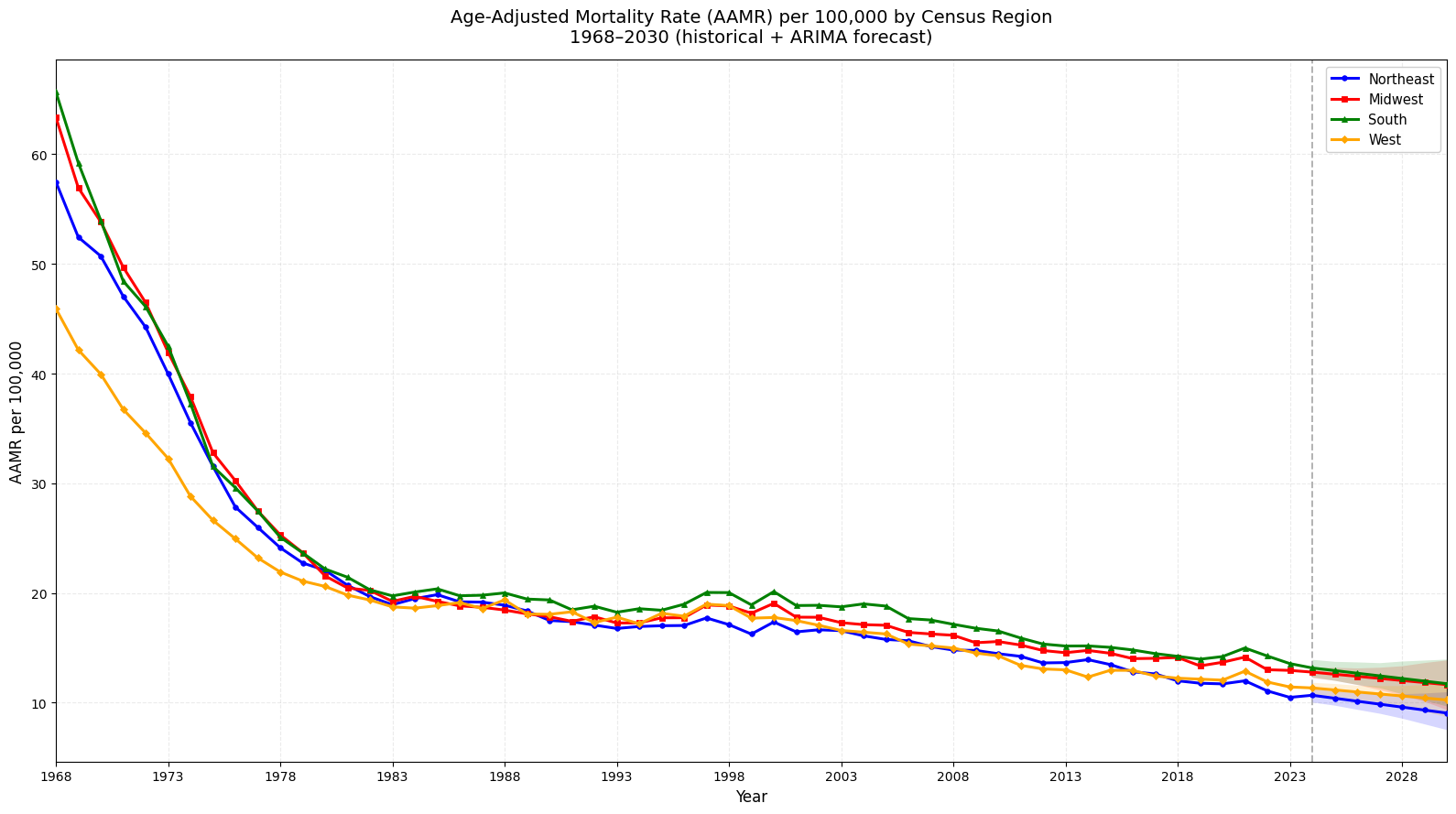


**Supplemental Figure 3.** Hemorrhagic Stroke AAMRs per 100,000 stratified by Census Region in adults in the United States, 1968­­–2023 and projections through 2030.


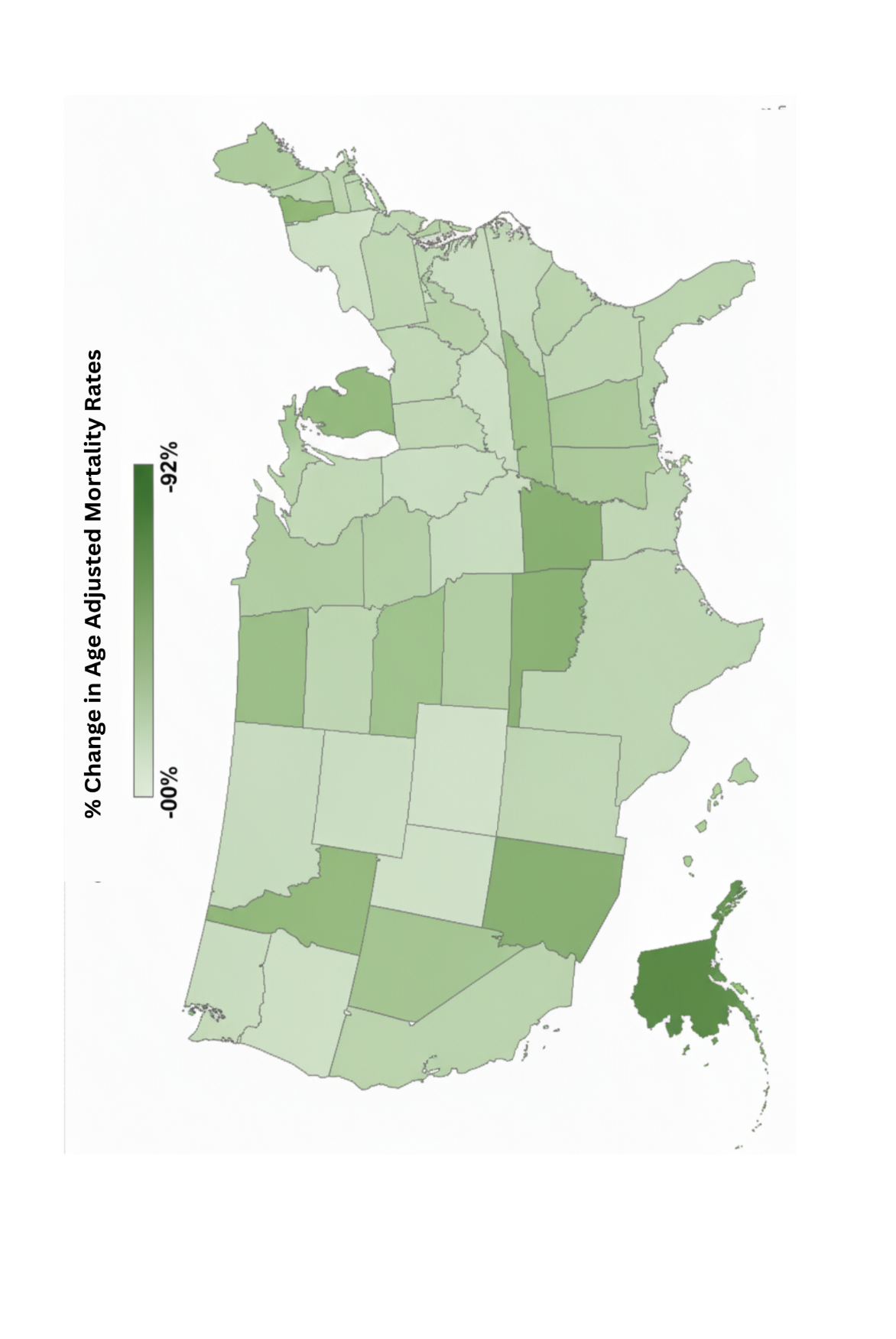


**Supplemental Figure 4.** Hemorrhagic Stroke percentage change (%) in AAMRs per 100,000 stratified by state in adults in the United States, 1968–­202

| **Supplemental Table 1, Hemorrhagic related mortalities in Adults, Stratified by Sex and Race, in the United States, 1968 to 2023** | | | | | | |
| --- | --- | --- | --- | --- | --- | --- |
| **Deaths** | | | | | | |
| **Year** | **Overall** | **Women** | **Men** | **Whites** | **Blacks** | **Population** |
| 1968 | 55670 | 29502 | 26168 | 47038 | 8259 | 107469000 |
| 1969 | 51749 | 27779 | 23970 | 43769 | 7619 | 108734000 |
| 1970 | 49851 | 26896 | 22955 | 42123 | 7351 | 109870505 |
| 1971 | 47077 | 25324 | 21753 | 40050 | 6644 | 111675000 |
| 1972 | 45478 | 24656 | 20822 | 38660 | 6436 | 114147000 |
| 1973 | 42457 | 23421 | 19036 | 36010 | 6055 | 116274000 |
| 1974 | 38759 | 21493 | 17266 | 32943 | 5478 | 118403000 |
| 1975 | 34714 | 19524 | 15190 | 29447 | 4903 | 120564000 |
| 1976 | 32440 | 18213 | 14227 | 27417 | 4664 | 122914000 |
| 1977 | 30688 | 17200 | 13488 | 25843 | 4478 | 125376000 |
| 1978 | 29040 | 16432 | 12608 | 24411 | 4266 | 127957000 |
| 1979 | 28111 | 15814 | 12297 | 23555 | 4193 | 130668000 |
| 1980 | 26901 | 15283 | 11618 | 22390 | 4119 | 132768638 |
| 1981 | 26303 | 15110 | 11193 | 22021 | 3881 | 136009066 |
| 1982 | 25874 | 14730 | 11144 | 21584 | 3888 | 138690289 |
| 1983 | 25361 | 14490 | 10871 | 21034 | 3873 | 141303090 |
| 1984 | 26295 | 15160 | 11135 | 21850 | 3948 | 143881783 |
| 1985 | 26818 | 15367 | 11451 | 22186 | 4061 | 146398041 |
| 1986 | 26770 | 15473 | 11297 | 22068 | 4113 | 149041120 |
| 1987 | 27090 | 15503 | 11587 | 22257 | 4142 | 151539755 |
| 1988 | 27663 | 15649 | 12014 | 22630 | 4356 | 153897145 |
| 1989 | 27162 | 15513 | 11649 | 22148 | 4290 | 156206784 |
| 1990 | 27083 | 15649 | 11434 | 22125 | 4183 | 157843690 |
| 1991 | 27172 | 15570 | 11602 | 22129 | 4169 | 161231105 |
| 1992 | 27746 | 15842 | 11904 | 22797 | 4067 | 163875074 |
| 1993 | 27855 | 15681 | 12174 | 22840 | 4132 | 166358177 |
| 1994 | 28420 | 16098 | 12322 | 23030 | 4390 | 168791121 |
| 1995 | 29385 | 16693 | 12692 | 24026 | 4306 | 171332456 |
| 1996 | 30236 | 17198 | 13038 | 24778 | 4361 | 173893568 |
| 1997 | 32575 | 18372 | 14203 | 26717 | 4628 | 176251758 |
| 1998 | 32847 | 18593 | 14254 | 27008 | 4601 | 178366837 |
| 1999 | 31733 | 18048 | 13685 | 26144 | 4325 | 180408769 |
| 2000 | 33692 | 19060 | 14632 | 27713 | 4689 | 181984640 |
| 2001 | 32514 | 18246 | 14268 | 26542 | 4583 | 184305128 |
| 2002 | 32939 | 18572 | 14367 | 27022 | 4513 | 186208028 |
| 2003 | 33008 | 18342 | 14666 | 26680 | 4836 | 188090429 |
| 2004 | 33385 | 18480 | 14905 | 27243 | 4737 | 190205384 |
| 2005 | 33620 | 18525 | 15095 | 27380 | 4786 | 192551384 |
| 2006 | 32780 | 18078 | 14702 | 26538 | 4707 | 195019359 |
| 2007 | 32914 | 18158 | 14756 | 26684 | 4712 | 197403777 |
| 2008 | 32993 | 18044 | 14949 | 26738 | 4686 | 199795090 |
| 2009 | 32795 | 17759 | 15036 | 26696 | 4566 | 202107016 |
| 2010 | 32893 | 17777 | 15116 | 26742 | 4495 | 203891983 |
| 2011 | 32502 | 17487 | 15015 | 26384 | 4464 | 206592936 |
| 2012 | 32108 | 17416 | 14692 | 26055 | 4375 | 208826037 |
| 2013 | 32482 | 17402 | 15080 | 26294 | 4486 | 211085314 |
| 2014 | 33096 | 17781 | 15315 | 26762 | 4578 | 213809280 |
| 2015 | 33603 | 18072 | 15531 | 27146 | 4565 | 216553817 |
| 2016 | 33460 | 17938 | 15522 | 26919 | 4503 | 218641417 |
| 2017 | 33668 | 17968 | 15700 | 26980 | 4647 | 221447331 |
| 2018 | 33724 | 17916 | 15808 | 26990 | 4655 | 223311190 |
| 2019 | 33538 | 17811 | 15727 | 26818 | 4625 | 224981167 |
| 2020 | 34294 | 17722 | 16572 | 27127 | 4903 | 226635013 |
| 2021 | 35229 | 18220 | 17009 | 27575 | 5203 | 228238412 |
| 2022 | 34160 | 17807 | 16353 | 26773 | 4993 | 229508599 |
| 2023 | 33121 | 17176 | 15945 | 26048 | 4776 | 231529762 |
| **Total** | **1845841** | **1018033** | **827808** | **1516877** | **266262** | **9544862264** |

| **Supplemental Table 2 Annual Percent Change (APC) and Average Annual Percentage Change (AAPC) of Hemorrhagic Stroke Age-Adjusted Mortality Rates per 100,000, in Adults, in the United States, 1968 to 2023** | |
| --- | --- |
| **Year Interval** | **APC/AAPC (95% CI)** |
| **Overall** | |
| 1968-1973 | -7.41* (-8.19 to -6.62) |
| 1973-1976 | -11.35* (-15.15 to -7.37) |
| 1976-1981 | -6.21* (-7.67 to -4.74) |
| 1981-1994 | -1.13* (-1.43 to -0.83) |
| 1994-1998 | 2.18 (-0.16 to 4.57) |
| 1998-2018 | -1.82* (-1.95 to -1.68) |
| 2018-2021 | 0.72 (-3.58 to 5.20) |
| 2021-2023 | -4.40* (-8.63 to 0.02) |
| **Female** | |
| 1968-1973 | -7.21* (-8.06 to -6.35) |
| 1973-1977 | -10.06* (-12.22 to -7.92) |
| 1977-1981 | -5.46* (-7.99 to -2.86) |
| 1981-1994 | -1.30* (-1.61 to -0.98) |
| 1994-1998 | 2.14 (-0.48 to 4.83) |
| 1998-2013 | -2.04* (-2.27 to -1.80) |
| 2013-2023 | -1.42* (-1.81 to -1.04) |
| **Male** | |
| 1968-1980 | -8.88* (-9.27 to -8.49) |
| 1980-1994 | -1.06* (-1.50 to -0.61) |
| 1994-1998 | 2.24 (-1.70 to 6.34) |
| 1998-2023 | -1.53* (-1.67 to -1.38) |
| **Younger Adults (25-44)** | |
| 1968-1972 | -3.14* (-5.12 to -1.11) |
| 1972-1980 | -6.95* (-7.89 to -6.00) |
| 1980-1997 | -1.84* (-2.14 to -1.55) |
| 1997-2013 | -3.01* (-3.38 to -2.65) |
| 2013-2023 | 0.53 (-0.26 to 1.32) |
| **Middle-Aged Adults (45-64)** | |
| 1968-1972 | -3.79* (-5.06 to -2.50) |
| 1972-1977 | -8.03* (-9.42 to -6.62) |
| 1977-1982 | -4.14* (-5.83 to -2.41) |
| 1982-1997 | -1.31* (-1.58 to -1.03) |
| 1997-2017 | -2.41* (-2.57 to -2.24) |
| 2017-2021 | 2.68 (-0.35 to 5.81) |
| 2021-2023 | -4.49 (-9.96 to 1.30) |
| **Older Adults (65+)** | |
| 1968-1973 | -8.77* (-9.81 to -7.72) |
| 1973-1976 | -12.66* (-17.75 to -7.24) |
| 1976-1981 | -6.83* (-8.82 to -4.79) |
| 1981-1993 | -0.86* (-1.32 to -0.40) |
| 1993-2000 | 1.95* (0.89 to 3.02) |
| 2000-2023 | -1.67* (-1.80 to -1.55) |
| **Black or African American** | |
| 1968-1977 | -9.14* (-9.73 to -8.54) |
| 1977-1981 | -4.63* (-8.64 to -0.45) |
| 1981-2003 | -1.27* (-1.48 to -1.06) |
| 2003-2016 | -2.83* (-3.30 to -2.36) |
| 2016-2023 | -0.04 (-1.12 to 1.04) |
| **White** | |
| 1968-1973 | -7.33* (-8.22 to -6.42) |
| 1973-1976 | -11.50* (-15.84 to -6.94) |
| 1976-1981 | -6.45* (-8.13 to -4.74) |
| 1981-1994 | -1.27* (-1.61 to -0.93) |
| 1994-1997 | 3.55 (-2.15 to 9.59) |
| 1997-2023 | -1.53* (-1.62 to -1.43) |
| **Hispanic** | |
| 1999-2018 | -2.17(-2.64 to -0.70) |
| 2018-2021 | 2.74(-4.08 to 4.23) |
| 2021-2023 | -5.11(-8.57 to 0.09) |
| **American Indian** | |
| 1999-2023 | -1.61(-2.10 to -1.08) |
| **Northeast** | |
| 1968-1972 | -6.07* (-7.49 to -4.64) |
| 1972-1977 | -10.55* (-12.12 to -8.95) |
| 1977-1982 | -4.68* (-6.64 to -2.69) |
| 1982-1993 | -1.36* (-1.88 to -0.83) |
| 1993-2003 | -0.31 (-0.91 to 0.29) |
| 2003-2023 | -2.03* (-2.21 to -1.84) |
| **Midwest** | |
| 1968-1973 | -7.57* (-8.38 to -6.76) |
| 1973-1976 | -11.13* (-15.06 to -7.02) |
| 1976-1981 | -7.43* (-8.97 to -5.86) |
| 1981-1994 | -1.26* (-1.63 to -0.89) |
| 1994-1997 | 3.53 (-1.98 to 9.35) |
| 1997-2023 | -1.46* (-1.56 to -1.36) |
| **South** | |
| 1968-1980 | -8.98* (-9.57 to -8.58) |
| 1980-1993 | -1.01* (-1.51 to -0.52) |
| 1993-2000 | 1.07 (-0.21 to 2.36) |
| 2000-2017 | -1.90* (-2.17 to -1.63) |
| 2017-2023 | -0.33 (-1.49 to 0.83) |
| **West** | |
| 1968-1979 | -7.17* (-7.46 to -6.76) |
| 1979-1994 | -1.02* (-1.33 to -0.70) |
| 1994-1997 | 3.18 (-2.94 to 9.68) |
| 1997-2014 | -2.32* (-2.53 to -2.10) |
| 2014-2023 | -0.84* (-1.36 to -0.32) |
| Hispanic and American Indian populations data was restricted to data from 1999 onwards | |

| **Supplemental Table 3 Overall and Sex‐Stratified Hemorrhagic Stroke related Age-Adjusted Mortality Rates per 100,000, in Adults, in the United States, 1968 to 2023** | | | |
| --- | --- | --- | --- |
| **Age-Adjusted Rate (95% CI)** | | | |
| **Year** | **Men** | **Women** | **Overall** |
| **1968** | 63.91 (63.06 - 64.75) | 56.68 (56.01 - 57.35) | 59.86 (59.34 - 60.39) |
| **1969** | 57.24 (56.45 - 58.03) | 51.73 (51.10 - 52.35) | 54.15 (53.67 - 54.64) |
| **1970** | 52.65 (51.93 - 53.37) | 48.25 (47.66 - 48.84) | 50.24 (49.78 - 50.70) |
| **1971** | 49.31 (48.60 - 50.01) | 44.33 (43.77 - 44.89) | 46.53 (46.09 - 46.96) |
| **1972** | 46.54 (45.86 - 47.21) | 42.04 (41.50 - 42.57) | 43.97 (43.55 - 44.39) |
| **1973** | 41.80 (41.17 - 42.44) | 39.03 (38.52 - 39.54) | 40.27 (39.87 - 40.66) |
| **1974** | 37.23 (36.64 - 37.82) | 34.79 (34.31 - 35.26) | 35.84 (35.47 - 36.21) |
| **1975** | 31.81 (31.27 - 32.34) | 30.61 (30.18 - 31.05) | 31.13 (30.79 - 31.47) |
| **1976** | 29.15 (28.65 - 29.66) | 27.92 (27.51 - 28.33) | 28.45 (28.14 - 28.77) |
| **1977** | 27.28 (26.79 - 27.76) | 25.66 (25.27 - 26.04) | 26.33 (26.03 - 26.63) |
| **1978** | 24.82 (24.36 - 25.28) | 23.93 (23.56 - 24.30) | 24.32 (24.03 - 24.60) |
| **1979** | 23.74 (23.30 - 24.18) | 22.37 (22.02 - 22.72) | 22.96 (22.69 - 23.23) |
| **1980** | 22.26 (21.83 - 22.69) | 21.36 (21.02 - 21.70) | 21.72 (21.45 - 21.98) |
| **1981** | 20.95 (20.54 - 21.35) | 20.58 (20.25 - 20.91) | 20.72 (20.46 - 20.97) |
| **1982** | 20.42 (20.02 - 20.82) | 19.63 (19.31 - 19.95) | 19.97 (19.73 - 20.22) |
| **1983** | 19.61 (19.22 - 19.99) | 18.94 (18.63 - 19.25) | 19.24 (19.00 - 19.48) |
| **1984** | 19.89 (19.50 - 20.27) | 19.44 (19.13 - 19.75) | 19.60 (19.35 - 19.84) |
| **1985** | 20.00 (19.62 - 20.39) | 19.33 (19.02 - 19.64) | 19.68 (19.44 - 19.92) |
| **1986** | 19.47 (19.10 - 19.85) | 19.10 (18.79 - 19.40) | 19.26 (19.03 - 19.50) |
| **1987** | 19.66 (19.29 - 20.03) | 18.75 (18.45 - 19.05) | 19.14 (18.91 - 19.38) |
| **1988** | 19.96 (19.58 - 20.33) | 18.62 (18.33 - 18.92) | 19.23 (19.00 - 19.46) |
| **1989** | 19.25 (18.89 - 19.62) | 18.15 (17.86 - 18.43) | 18.60 (18.38 - 18.83) |
| **1990** | 18.54 (18.19 - 18.90) | 18.08 (17.79 - 18.36) | 18.31 (18.09 - 18.53) |
| **1991** | 18.38 (18.03 - 18.73) | 17.56 (17.28 - 17.84) | 17.93 (17.71 - 18.14) |
| **1992** | 18.46 (18.12 - 18.80) | 17.46 (17.19 - 17.74) | 17.90 (17.69 - 18.11) |
| **1993** | 18.44 (18.10 - 18.78) | 16.96 (16.69 - 17.22) | 17.60 (17.40 - 17.81) |
| **1994** | 18.32 (17.99 - 18.65) | 17.11 (16.85 - 17.38) | 17.64 (17.44 - 17.85) |
| **1995** | 18.53 (18.20 - 18.86) | 17.39 (17.12 - 17.65) | 17.90 (17.70 - 18.11) |
| **1996** | 18.62 (18.29 - 18.95) | 17.58 (17.31 - 17.84) | 18.06 (17.85 - 18.26) |
| **1997** | 19.99 (19.65 - 20.32) | 18.39 (18.12 - 18.66) | 19.09 (18.88 - 19.29) |
| **1998** | 19.67 (19.34 - 19.99) | 18.27 (18.01 - 18.54) | 18.90 (18.69 - 19.10) |
| **1999** | 18.60 (18.28 - 18.91) | 17.44 (17.18 - 17.70) | 17.96 (17.77 - 18.16) |
| **2000** | 19.73 (19.41 - 20.06) | 18.16 (17.90 - 18.42) | 18.81 (18.61 - 19.01) |
| **2001** | 18.77 (18.46 - 19.09) | 17.12 (16.87 - 17.37) | 17.84 (17.64 - 18.03) |
| **2002** | 18.60 (18.29 - 18.91) | 17.20 (16.95 - 17.44) | 17.81 (17.61 - 18.00) |
| **2003** | 18.62 (18.31 - 18.93) | 16.77 (16.52 - 17.01) | 17.53 (17.34 - 17.72) |
| **2004** | 18.55 (18.25 - 18.86) | 16.65 (16.41 - 16.89) | 17.47 (17.28 - 17.66) |
| **2005** | 18.39 (18.09 - 18.69) | 16.41 (16.17 - 16.65) | 17.28 (17.09 - 17.46) |
| **2006** | 17.49 (17.20 - 17.77) | 15.75 (15.52 - 15.98) | 16.49 (16.31 - 16.67) |
| **2007** | 17.16 (16.88 - 17.44) | 15.56 (15.33 - 15.79) | 16.29 (16.11 - 16.46) |
| **2008** | 17.09 (16.81 - 17.37) | 15.19 (14.96 - 15.41) | 16.02 (15.84 - 16.19) |
| **2009** | 16.81 (16.53 - 17.08) | 14.72 (14.50 - 14.94) | 15.66 (15.49 - 15.83) |
| **2010** | 16.64 (16.37 - 16.91) | 14.52 (14.30 - 14.74) | 15.47 (15.30 - 15.64) |
| **2011** | 16.02 (15.76 - 16.28) | 14.02 (13.81 - 14.23) | 14.93 (14.76 - 15.09) |
| **2012** | 15.31 (15.05 - 15.56) | 13.68 (13.47 - 13.88) | 14.42 (14.26 - 14.58) |
| **2013** | 15.32 (15.07 - 15.57) | 13.38 (13.17 - 13.58) | 14.28 (14.12 - 14.43) |
| **2014** | 15.21 (14.96 - 15.45) | 13.40 (13.20 - 13.60) | 14.23 (14.07 - 14.38) |
| **2015** | 15.08 (14.84 - 15.33) | 13.40 (13.20 - 13.60) | 14.18 (14.02 - 14.33) |
| **2016** | 14.74 (14.51 - 14.98) | 13.06 (12.87 - 13.26) | 13.86 (13.71 - 14.01) |
| **2017** | 14.54 (14.31 - 14.77) | 12.80 (12.61 - 12.99) | 13.59 (13.44 - 13.74) |
| **2018** | 14.29 (14.06 - 14.52) | 12.54 (12.35 - 12.73) | 13.34 (13.20 - 13.49) |
| **2019** | 13.96 (13.74 - 14.19) | 12.26 (12.08 - 12.45) | 13.04 (12.90 - 13.18) |
| **2020** | 14.41 (14.18 - 14.63) | 12.09 (11.91 - 12.27) | 13.17 (13.03 - 13.32) |
| **2021** | 14.88 (14.65 - 15.10) | 12.82 (12.63 - 13.01) | 13.81 (13.66 - 13.96) |
| **2022** | 13.96 (13.74 - 14.18) | 12.01 (11.83 - 12.19) | 12.89 (12.75 - 13.03) |
| **2023** | 13.37 (13.15 - 13.58) | 11.54 (11.37 - 11.72) | 12.39 (12.26 - 12.53) |

| **Supplemental Table 4 Race‐Stratified Hemorrhagic Stroke Related Age-Adjusted Mortality Rates per 100,000 in Adults, in the United States, 1968 to 2023** | | |
| --- | --- | --- |
| **Age-Adjusted Rate (95% CI)** | | |
| **Year** | **Whites** | **Blacks** |
| **1968** | 56.33 (55.80–56.87) | 96.14 (93.92–98.37) |
| **1969** | 50.97 (50.47–51.47) | 86.75 (84.67–88.82) |
| **1970** | 47.28 (46.82–47.75) | 80.71 (78.77–82.65) |
| **1971** | 44.01 (43.56–44.45) | 71.06 (69.26–72.85) |
| **1972** | 41.57 (41.14–41.99) | 67.19 (65.47–68.91) |
| **1973** | 37.97 (37.57–38.38) | 61.74 (60.11–63.36) |
| **1974** | 33.87 (33.49–34.24) | 54.79 (53.28–56.30) |
| **1975** | 29.37 (29.02–29.71) | 47.74 (46.35–49.12) |
| **1976** | 26.77 (26.44–27.09) | 44.07 (42.77–45.38) |
| **1977** | 24.69 (24.38–25.00) | 41.37 (40.12–42.62) |
| **1978** | 22.77 (22.47–23.06) | 38.38 (37.19–39.56) |
| **1979** | 21.40 (21.12–21.68) | 36.96 (35.81–38.11) |
| **1980** | 20.14 (19.87–20.41) | 36.19 (35.05–37.33) |
| **1981** | 19.34 (19.08–19.60) | 33.04 (31.97–34.11) |
| **1982** | 18.58 (18.33–18.83) | 32.58 (31.53–33.64) |
| **1983** | 17.80 (17.56–18.05) | 32.02 (30.99–33.06) |
| **1984** | 18.20 (17.96–18.45) | 31.75 (30.73–32.76) |
| **1985** | 18.17 (17.93–18.41) | 32.57 (31.54–33.59) |
| **1986** | 17.75 (17.51–17.99) | 31.94 (30.94–32.93) |
| **1987** | 17.58 (17.35–17.81) | 31.67 (30.69–32.66) |
| **1988** | 17.62 (17.39–17.86) | 32.63 (31.64–33.62) |
| **1989** | 16.98 (16.76–17.21) | 31.79 (30.81–32.76) |
| **1990** | 16.78 (16.55–17.00) | 30.40 (29.45–31.34) |
| **1991** | 16.38 (16.16–16.59) | 29.68 (28.76–30.60) |
| **1992** | 16.51 (16.30–16.73) | 28.01 (27.13–28.89) |
| **1993** | 16.24 (16.02–16.45) | 27.91 (27.05–28.78) |
| **1994** | 16.11 (15.90–16.31) | 28.85 (27.98–29.72) |
| **1995** | 16.52 (16.31–16.73) | 27.62 (26.78–28.46) |
| **1996** | 16.72 (16.51–16.93) | 27.39 (26.56–28.21) |
| **1997** | 17.70 (17.49–17.91) | 28.31 (27.48–29.14) |
| **1998** | 17.60 (17.39–17.81) | 27.55 (26.74–28.36) |
| **1999** | 16.75 (16.54–16.95) | 25.47 (24.69–26.24) |
| **2000** | 17.61 (17.40–17.81) | 27.18 (26.38–27.97) |
| **2001** | 16.60 (16.40–16.80) | 25.82 (25.06–26.58) |
| **2002** | 16.65 (16.45–16.85) | 24.92 (24.18–25.66) |
| **2003** | 16.24 (16.04–16.43) | 26.12 (25.36–26.87) |
| **2004** | 16.36 (16.16–16.55) | 24.93 (24.20–25.66) |
| **2005** | 16.16 (15.97–16.36) | 24.48 (23.77–25.20) |
| **2006** | 15.38 (15.19–15.56) | 23.45 (22.76–24.14) |
| **2007** | 15.26 (15.08–15.44) | 22.97 (22.30–23.65) |
| **2008** | 15.01 (14.83–15.19) | 22.30 (21.64–22.96) |
| **2009** | 14.75 (14.58–14.93) | 21.16 (20.53–21.80) |
| **2010** | 14.59 (14.42–14.77) | 20.40 (19.78–21.02) |
| **2011** | 14.11 (13.94–14.29) | 19.87 (19.26–20.47) |
| **2012** | 13.68 (13.51–13.85) | 18.93 (18.35–19.51) |
| **2013** | 13.51 (13.35–13.68) | 19.03 (18.45–19.60) |
| **2014** | 13.53 (13.37–13.69) | 18.92 (18.35–19.48) |
| **2015** | 13.50 (13.34–13.67) | 18.36 (17.81–18.91) |
| **2016** | 13.18 (13.02–13.34) | 17.63 (17.10–18.16) |
| **2017** | 12.91 (12.76–13.07) | 17.66 (17.14–18.18) |
| **2018** | 12.72 (12.56–12.87) | 17.38 (16.87–17.89) |
| **2019** | 12.42 (12.27–12.57) | 16.90 (16.40–17.40) |
| **2020** | 12.46 (12.31–12.61) | 17.33 (16.83–17.83) |
| **2021** | 13.08 (12.92–13.23) | 18.86 (18.33–19.39) |
| **2022** | 12.22 (12.07–12.37) | 17.70 (17.20–18.20) |
| **2023** | 11.85 (11.70–12.00) | 16.63 (16.15–17.12) |

| **Supplemental Table 5 Race‐Stratified Hemorrhagic Stroke Related Age-Adjusted Mortality Rates per 100,000 in Adults, in the United States, 1968 to 2023** | | |
| --- | --- | --- |
| **Age-Adjusted Rate (95% CI)** | | |
| **Year** | **Hispanic** | **American Indian** |
| **1999** | 18.14 (17.25–19.03) | 17.15(14.1–20.19) |
| **2000** | 17.7 (16.84–18.56) | 16.81(13.89–19.73) |
| **2001** | 17.11 (16.3–17.93) | 18.79(15.77–21.81) |
| **2002** | 16.64 (15.85–17.43) | 15.65(12.95–18.36) |
| **2003** | 16 (15.25–16.74) | 17.72(14.84–20.59) |
| **2004** | 17.33 (16.57–18.09) | 16.68(13.95–19.41) |
| **2005** | 16.65 (15.93–17.38) | 17.13(14.42–19.83) |
| **2006** | 16.24 (15.55–16.94) | 14.46(11.97–16.95) |
| **2007** | 15.73 (15.06–16.4) | 14.39(11.99–16.78) |
| **2008** | 14.92 (14.28–15.56) | 15.53(13.01–18.05) |
| **2009** | 14.08 (13.48–14.68) | 17.04(14.41–19.67) |
| **2010** | 14.63 (14.02–15.23) | 13.87(11.55–16.19) |
| **2011** | 13.88 (13.31–14.45) | 14.27(12.05–16.48) |
| **2012** | 13.2 (12.66–13.74) | 14.58(12.3–16.85) |
| **2013** | 13.78 (13.24–14.32) | 13.41(11.27–15.55) |
| **2014** | 12.6 (12.1–13.1) | 13.61(11.56–15.66) |
| **2015** | 12.91 (12.42–13.41) | 13.45(11.45–15.45) |
| **2016** | 12.95 (12.46–13.43) | 13.39(11.44–15.35) |
| **2017** | 12.35 (11.89–12.8) | 14.06(12.1–16.01) |
| **2018** | 11.93 (11.48–12.37) | 12.5(10.69–14.3) |
| **2019** | 12.31 (11.88–12.75) | 11.94(10.21–13.67) |
| **2020** | 12.61 (12.18–13.04) | 13.24(11.46–15.02) |
| **2021** | 13.06 (12.62–13.49) | 13.94(12.03–15.86) |
| **2022** | 12.5 (12.08–12.91) | 13.11(11.28–14.94) |
| **2023** | 11.69 (11.29–12.09) | 10.22(8.66–11.78) |
| Hispanic and American Indian populations data was restricted to data from 1999 onwards. | | |

| **Supplemental Table 6, Hemorrhagic Stroke related Age-Adjusted Mortality Rates per 100,000, Stratified by States, in Adults, in the United States, 1968 to 2023** | | | |
| --- | --- | --- | --- |
| **State** | **AAMR (1968)** | **AAMR (2023)** | **% Change (1968–2023)** |
| Alabama | 82.36 | 16.97 | -79.4 |
| Alaska | 28.92 | 12.38 | -57.19 |
| Arizona | 41.37 | 8.5 | -79.45 |
| Arkansas | 71.61 | 19.73 | -72.45 |
| California | 44.99 | 12 | -73.33 |
| Colorado | 45.34 | 11.23 | -75.23 |
| Connecticut | 60.33 | 8.89 | -85.26 |
| Delaware | 44.09 | 17.95 | -59.29 |
| District of Columbia | 56.04 | 12.74 | -77.27 |
| Florida | 46.78 | 10.35 | -77.88 |
| Georgia | 99.93 | 15.74 | -84.25 |
| Hawaii | 51.87 | 14.29 | -72.45 |
| Idaho | 46.13 | 10.22 | -77.85 |
| Illinois | 71.63 | 12.95 | -81.92 |
| Indiana | 77.49 | 12.99 | -83.24 |
| Iowa | 58.52 | 11.2 | -80.86 |
| Kansas | 62.07 | 15.06 | -75.74 |
| Kentucky | 64.24 | 16.92 | -73.66 |
| Louisiana | 63.39 | 15.53 | -75.5 |
| Maine | 71.98 | 3.92 | -94.55 |
| Maryland | 46.73 | 12.68 | -72.87 |
| Massachusetts | 70.05 | 9.87 | -85.91 |
| Michigan | 56.14 | 12.24 | -78.2 |
| Minnesota | 57.53 | 10.58 | -81.61 |
| Mississippi | 78.84 | 16.85 | -78.63 |
| Missouri | 60.22 | 16.18 | -73.13 |
| Montana | 47.41 | 10.03 | -78.84 |
| Nebraska | 57.08 | 13.22 | -76.84 |
| Nevada | 61.27 | 13.47 | -78.02 |
| New Hampshire | 68.75 | 10.97 | -84.04 |
| New Jersey | 49.83 | 12.79 | -74.33 |
| New Mexico | 59.1 | 9.31 | -84.25 |
| New York | 54.84 | 9.74 | -82.24 |
| North Carolina | 73.15 | 11.46 | -84.33 |
| North Dakota | 60.7 | 12.96 | -78.65 |
| Ohio | 64.38 | 13.68 | -78.75 |
| Oklahoma | 64.78 | 10.21 | -84.24 |
| Oregon | 47.01 | 13.42 | -71.45 |
| Pennsylvania | 55.71 | 11.87 | -78.69 |
| Rhode Island | 58.62 | 8.66 | -85.23 |
| South Carolina | 100.03 | 15.63 | -84.37 |
| South Dakota | 55.38 | 13.69 | -75.28 |
| Tennessee | 62.56 | 16.82 | -73.11 |
| Texas | 58.15 | 14.9 | -74.38 |
| Utah | 39.56 | 9.02 | -77.2 |
| Vermont | 64.13 | 6.96 | -89.15 |
| Virginia | 58.1 | 11.35 | -80.46 |
| Washington | 47.66 | 11.47 | -75.93 |
| West Virginia | 70.36 | 8.2 | -88.35 |
| Wisconsin | 54.14 | 11.22 | -79.28 |
| Wyoming | 61.81 | 13.69 | -77.85 |

| **Supplemental Table 7, Hemorrhagic Stroke related Age-Adjusted Mortality Rates per 100,000, Stratified by Census Region, in Adults, in the United States, 1968 to 2023** | | | | |
| --- | --- | --- | --- | --- |
|  | **Census Region: NorthEast** | **Census Region: Midwest** | **Census Region:  South** | **Census Region:  West** |
| **Year** | **Age-Adjusted Rate (95% CI)** | **Age-Adjusted Rate (95% CI)** | **Age-Adjusted Rate (95% CI)** | **Age-Adjusted Rate (95% CI)** |
| **1968** | 57.48 (56.47–58.49) | 63.36 (62.36–64.36) | 65.68 (64.67–66.70) | 45.92 (44.76–47.07) |
| **1969** | 52.43 (51.47–53.38) | 56.93 (56.00–57.87) | 59.20 (58.26–60.15) | 42.16 (41.08–43.23) |
| **1970** | 50.71 (49.78–51.63) | 53.83 (52.94–54.73) | 53.97 (53.09–54.85) | 39.95 (38.93–40.98) |
| **1971** | 47.05 (46.18–47.92) | 49.70 (48.85–50.54) | 48.43 (47.61–49.24) | 36.74 (35.78–37.70) |
| **1972** | 44.23 (43.40–45.07) | 46.47 (45.66–47.27) | 46.10 (45.32–46.88) | 34.58 (33.67–35.50) |
| **1973** | 39.98 (39.19–40.76) | 41.95 (41.19–42.71) | 42.51 (41.78–43.24) | 32.27 (31.40–33.14) |
| **1974** | 35.52 (34.79–36.25) | 37.89 (37.18–38.61) | 37.28 (36.60–37.95) | 28.81 (28.00–29.61) |
| **1975** | 31.54 (30.86–32.22) | 32.79 (32.13–33.44) | 31.54 (30.93–32.14) | 26.63 (25.87–27.39) |
| **1976** | 27.84 (27.20–28.48) | 30.24 (29.61–30.87) | 29.60 (29.02–30.18) | 24.94 (24.21–25.66) |
| **1977** | 25.98 (25.37–26.59) | 27.48 (26.88–28.07) | 27.47 (26.91–28.02) | 23.20 (22.51–23.88) |
| **1978** | 24.13 (23.55–24.71) | 25.30 (24.74–25.86) | 25.09 (24.57–25.60) | 21.92 (21.27–22.58) |
| **1979** | 22.74 (22.18–23.30) | 23.68 (23.14–24.22) | 23.66 (23.17–24.15) | 21.09 (20.46–21.72) |
| **1980** | 22.07 (21.52–22.62) | 21.56 (21.05–22.08) | 22.19 (21.72–22.66) | 20.60 (19.98–21.22) |
| **1981** | 20.71 (20.18–21.24) | 20.46 (19.97–20.95) | 21.44 (20.98–21.90) | 19.81 (19.22–20.41) |
| **1982** | 19.67 (19.16–20.18) | 20.23 (19.74–20.72) | 20.29 (19.86–20.73) | 19.36 (18.78–19.94) |
| **1983** | 18.94 (18.44–19.43) | 19.24 (18.77–19.72) | 19.74 (19.32–20.16) | 18.74 (18.17–19.30) |
| **1984** | 19.49 (18.99–19.99) | 19.70 (19.22–20.17) | 20.09 (19.67–20.51) | 18.62 (18.07–19.18) |
| **1985** | 19.85 (19.35–20.35) | 19.24 (18.77–19.71) | 20.38 (19.96–20.80) | 18.85 (18.30–19.41) |
| **1986** | 19.20 (18.71–19.69) | 18.83 (18.38–19.29) | 19.75 (19.34–20.16) | 19.13 (18.58–19.68) |
| **1987** | 19.17 (18.68–19.65) | 18.70 (18.25–19.15) | 19.80 (19.39–20.20) | 18.59 (18.06–19.12) |
| **1988** | 18.89 (18.41–19.37) | 18.45 (18.00–18.90) | 20.01 (19.61–20.42) | 19.37 (18.83–19.90) |
| **1989** | 18.37 (17.90–18.85) | 18.10 (17.66–18.54) | 19.45 (19.05–19.84) | 18.09 (17.58–18.60) |
| **1990** | 17.50 (17.04–17.96) | 17.84 (17.40–18.27) | 19.37 (18.98–19.75) | 18.06 (17.55–18.56) |
| **1991** | 17.39 (16.94–17.84) | 17.42 (16.99–17.84) | 18.47 (18.09–18.84) | 18.29 (17.79–18.79) |
| **1992** | 17.08 (16.63–17.52) | 17.84 (17.42–18.27) | 18.80 (18.43–19.17) | 17.31 (16.83–17.79) |
| **1993** | 16.77 (16.33–17.21) | 17.27 (16.86–17.69) | 18.25 (17.89–18.61) | 17.80 (17.33–18.28) |
| **1994** | 16.95 (16.52–17.39) | 17.28 (16.87–17.69) | 18.57 (18.21–18.93) | 17.20 (16.74–17.66) |
| **1995** | 17.02 (16.58–17.45) | 17.74 (17.33–18.15) | 18.43 (18.08–18.79) | 18.15 (17.68–18.61) |
| **1996** | 17.04 (16.61–17.47) | 17.75 (17.33–18.16) | 18.98 (18.63–19.34) | 17.89 (17.43–18.35) |
| **1997** | 17.73 (17.30–18.17) | 18.91 (18.49–19.34) | 20.05 (19.69–20.41) | 18.99 (18.52–19.45) |
| **1998** | 17.12 (16.70–17.55) | 18.82 (18.40–19.24) | 20.04 (19.68–20.39) | 18.85 (18.39–19.30) |
| **1999** | 16.26 (15.85–16.67) | 18.16 (17.75–18.56) | 18.92 (18.58–19.26) | 17.71 (17.27–18.15) |
| **2000** | 17.36 (16.94–17.79) | 19.06 (18.64–19.47) | 20.13 (19.78–20.48) | 17.77 (17.33–18.20) |
| **2001** | 16.46 (16.05–16.87) | 17.80 (17.40–18.20) | 18.86 (18.53–19.20) | 17.49 (17.07–17.92) |
| **2002** | 16.65 (16.24–17.05) | 17.79 (17.40–18.19) | 18.88 (18.55–19.22) | 17.06 (16.64–17.47) |
| **2003** | 16.57 (16.17–16.98) | 17.30 (16.91–17.69) | 18.74 (18.41–19.07) | 16.59 (16.18–16.99) |
| **2004** | 16.10 (15.71–16.50) | 17.12 (16.73–17.50) | 19.01 (18.68–19.34) | 16.43 (16.03–16.83) |
| **2005** | 15.77 (15.38–16.16) | 17.06 (16.68–17.45) | 18.81 (18.48–19.13) | 16.27 (15.88–16.66) |
| **2006** | 15.65 (15.26–16.04) | 16.41 (16.04–16.78) | 17.67 (17.35–17.98) | 15.35 (14.98–15.73) |
| **2007** | 15.12 (14.74–15.51) | 16.27 (15.91–16.64) | 17.54 (17.23–17.84) | 15.18 (14.81–15.55) |
| **2008** | 14.80 (14.42–15.17) | 16.15 (15.78–16.51) | 17.15 (16.85–17.45) | 14.99 (14.63–15.36) |
| **2009** | 14.77 (14.40–15.14) | 15.47 (15.12–15.83) | 16.79 (16.49–17.08) | 14.53 (14.17–14.88) |
| **2010** | 14.47 (14.10–14.83) | 15.58 (15.22–15.93) | 16.54 (16.25–16.83) | 14.28 (13.93–14.63) |
| **2011** | 14.23 (13.87–14.59) | 15.26 (14.91–15.61) | 15.90 (15.62–16.18) | 13.41 (13.08–13.74) |
| **2012** | 13.63 (13.27–13.98) | 14.76 (14.42–15.10) | 15.35 (15.08–15.62) | 13.08 (12.75–13.40) |
| **2013** | 13.66 (13.30–14.01) | 14.56 (14.23–14.90) | 15.17 (14.90–15.43) | 13.00 (12.68–13.32) |
| **2014** | 13.93 (13.57–14.28) | 14.77 (14.43–15.10) | 15.18 (14.91–15.44) | 12.34 (12.03–12.64) |
| **2015** | 13.48 (13.13–13.82) | 14.51 (14.18–14.84) | 15.05 (14.79–15.31) | 12.96 (12.64–13.27) |
| **2016** | 12.82 (12.48–13.16) | 14.02 (13.69–14.35) | 14.81 (14.56–15.07) | 12.95 (12.64–13.25) |
| **2017** | 12.63 (12.30–12.95) | 14.05 (13.73–14.38) | 14.48 (14.23–14.73) | 12.44 (12.14–12.73) |
| **2018** | 12.00 (11.68–12.32) | 14.13 (13.81–14.45) | 14.23 (13.98–14.47) | 12.24 (11.94–12.53) |
| **2019** | 11.78 (11.46–12.09) | 13.36 (13.05–13.67) | 13.97 (13.73–14.20) | 12.15 (11.86–12.43) |
| **2020** | 11.72 (11.41–12.03) | 13.69 (13.38–14.01) | 14.21 (13.97–14.45) | 12.06 (11.77–12.34) |
| **2021** | 12.00 (11.69–12.32) | 14.16 (13.83–14.48) | 14.99 (14.74–15.24) | 12.88 (12.58–13.18) |
| **2022** | 11.08 (10.78–11.38) | 13.01 (12.71–13.32) | 14.26 (14.02–14.49) | 11.89 (11.61–12.17) |
| **2023** | 10.49 (10.20–10.78) | 12.95 (12.64–13.25) | 13.57 (13.34–13.80) | 11.44 (11.16–11.71) |

| **Supplemental Table 8 Age‐Stratified Hemorrhagic Stroke related Age-Adjusted Mortality Rates per 100,000, in Adults, in the United States, 1968 to 2023** | | | |
| --- | --- | --- | --- |
| **Age-Adjusted Rate (95% CI)** | | | |
| **Year** | **Younger Adults (25-44)** | **Middle-Aged Adults (45-64)** | **Older Adults (65+)** |
| **1968** | 8.53 (8.26–8.80) | 36.89 (36.30–37.47) | 221.34 (218.94–223.73) |
| **1969** | 8.25 (7.99–8.52) | 34.14 (33.58–34.69) | 197.63 (195.41–199.85) |
| **1970** | 7.95 (7.69–8.21) | 33.77 (33.22–34.32) | 178.96 (176.93–180.99) |
| **1971** | 7.73 (7.47–7.98) | 32.37 (31.84–32.91) | 162.94 (161.01–164.87) |
| **1972** | 7.44 (7.19–7.70) | 31.19 (30.67–31.72) | 152.61 (150.77–154.45) |
| **1973** | 7.08 (6.84–7.33) | 28.84 (28.34–29.34) | 138.65 (136.93–140.38) |
| **1974** | 6.51 (6.27–6.75) | 26.43 (25.95–26.90) | 121.60 (120.01–123.18) |
| **1975** | 5.90 (5.67–6.12) | 23.81 (23.36–24.26) | 103.53 (102.10–104.96) |
| **1976** | 5.73 (5.51–5.95) | 22.31 (21.87–22.75) | 92.86 (91.53–94.20) |
| **1977** | 5.21 (5.00–5.41) | 20.53 (20.11–20.95) | 86.36 (85.09–87.62) |
| **1978** | 4.92 (4.72–5.12) | 19.79 (19.37–20.20) | 78.04 (76.86–79.23) |
| **1979** | 4.49 (4.31–4.68) | 18.85 (18.45–19.25) | 73.75 (72.62–74.88) |
| **1980** | 4.38 (4.20–4.56) | 17.86 (17.47–18.25) | 69.39 (68.30–70.48) |
| **1981** | 4.38 (4.21–4.56) | 17.41 (17.02–17.79) | 65.07 (64.04–66.11) |
| **1982** | 3.99 (3.83–4.16) | 16.81 (16.43–17.19) | 63.24 (62.24–64.25) |
| **1983** | 3.79 (3.63–3.95) | 16.37 (16.00–16.75) | 60.75 (59.78–61.72) |
| **1984** | 3.87 (3.71–4.03) | 16.30 (15.92–16.67) | 62.49 (61.52–63.46) |
| **1985** | 3.75 (3.60–3.90) | 15.75 (15.39–16.12) | 64.19 (63.22–65.17) |
| **1986** | 3.76 (3.61–3.91) | 15.64 (15.27–16.00) | 62.22 (61.27–63.16) |
| **1987** | 3.76 (3.61–3.90) | 15.63 (15.26–15.99) | 61.63 (60.70–62.57) |
| **1988** | 3.62 (3.48–3.76) | 15.62 (15.26–15.99) | 62.42 (61.49–63.34) |
| **1989** | 3.50 (3.36–3.64) | 15.01 (14.66–15.36) | 60.55 (59.64–61.45) |
| **1990** | 3.43 (3.29–3.56) | 14.89 (14.54–15.24) | 59.44 (58.55–60.33) |
| **1991** | 3.33 (3.20–3.46) | 14.48 (14.13–14.82) | 58.44 (57.57–59.31) |
| **1992** | 3.34 (3.21–3.47) | 14.21 (13.88–14.55) | 58.71 (57.85–59.57) |
| **1993** | 3.19 (3.07–3.32) | 14.01 (13.69–14.34) | 57.91 (57.07–58.75) |
| **1994** | 3.36 (3.23–3.49) | 14.05 (13.73–14.37) | 57.65 (56.82–58.48) |
| **1995** | 3.28 (3.15–3.40) | 13.97 (13.65–14.28) | 59.34 (58.50–60.18) |
| **1996** | 3.10 (2.98–3.22) | 13.97 (13.65–14.28) | 60.54 (59.70–61.38) |
| **1997** | 3.16 (3.04–3.28) | 13.98 (13.67–14.28) | 65.63 (64.77–66.50) |
| **1998** | 3.01 (2.89–3.13) | 13.50 (13.21–13.80) | 65.86 (65.00–66.73) |
| **1999** | 2.85 (2.73–2.96) | 12.39 (12.11–12.67) | 63.43 (62.59–64.27) |
| **2000** | 2.79 (2.68–2.90) | 13.13 (12.84–13.41) | 66.60 (65.74–67.46) |
| **2001** | 2.68 (2.57–2.79) | 12.07 (11.80–12.34) | 63.72 (62.88–64.55) |
| **2002** | 2.58 (2.47–2.69) | 11.77 (11.51–12.03) | 64.34 (63.50–65.17) |
| **2003** | 2.58 (2.47–2.69) | 11.72 (11.46–11.97) | 63.00 (62.18–63.82) |
| **2004** | 2.53 (2.42–2.64) | 11.70 (11.45–11.95) | 62.88 (62.07–63.70) |
| **2005** | 2.47 (2.36–2.58) | 11.50 (11.26–11.74) | 62.36 (61.55–63.16) |
| **2006** | 2.32 (2.21–2.42) | 11.24 (11.00–11.48) | 59.16 (58.38–59.94) |
| **2007** | 2.26 (2.16–2.37) | 10.90 (10.67–11.14) | 58.83 (58.06–59.60) |
| **2008** | 2.21 (2.11–2.31) | 10.32 (10.10–10.54) | 58.61 (57.85–59.37) |
| **2009** | 2.10 (2.00–2.20) | 10.18 (9.96–10.40) | 57.29 (56.54–58.03) |
| **2010** | 2.10 (2.00–2.20) | 9.80 (9.59–10.01) | 56.97 (56.23–57.71) |
| **2011** | 1.99 (1.89–2.09) | 9.60 (9.39–9.81) | 54.81 (54.09–55.53) |
| **2012** | 1.94 (1.84–2.03) | 9.25 (9.04–9.45) | 52.97 (52.28–53.67) |
| **2013** | 1.84 (1.74–1.93) | 9.10 (8.90–9.31) | 52.72 (52.03–53.40) |
| **2014** | 1.89 (1.79–1.99) | 8.96 (8.76–9.16) | 52.59 (51.92–53.27) |
| **2015** | 1.95 (1.85–2.04) | 8.78 (8.58–8.98) | 52.52 (51.85–53.18) |
| **2016** | 1.99 (1.89–2.09) | 8.76 (8.57–8.96) | 50.83 (50.18–51.48) |
| **2017** | 1.89 (1.80–1.99) | 8.50 (8.31–8.70) | 50.14 (49.51–50.78) |
| **2018** | 1.73 (1.64–1.82) | 8.53 (8.33–8.72) | 49.20 (48.58–49.82) |
| **2019** | 1.78 (1.69–1.87) | 8.45 (8.25–8.64) | 47.68 (47.08–48.28) |
| **2020** | 2.00 (1.90–2.10) | 9.35 (9.14–9.55) | 46.26 (45.68–46.85) |
| **2021** | 2.10 (2.00–2.20) | 9.30 (9.10–9.51) | 49.36 (48.74–49.97) |
| **2022** | 2.05 (1.96–2.15) | 8.87 (8.66–9.07) | 45.52 (44.95–46.10) |
| **2023** | 1.89 (1.80–1.98) | 8.49 (8.29–8.69) | 44.03 (43.47–44.59) |
